# Prevention of ribosome collision-induced neuromuscular degeneration by SARS CoV-2–encoded Nsp1

**DOI:** 10.1073/pnas.2202322119

**Published:** 2022-09-28

**Authors:** Xingjun Wang, Suman Rimal, Ishaq Tantray, Ji Geng, Sunil Bhurtel, Tejinder Pal Khaket, Wen Li, Zhe Han, Bingwei Lu

**Affiliations:** ^a^Department of Pathology, Stanford University School of Medicine, Stanford, CA 94350;; ^b^Center for Precision Disease Modeling, Department of Medicine, University of Maryland School of Medicine, Baltimore, MD 21201;; ^c^Programs of Neuroscience and Cancer Biology, Stanford University School of Medicine, Stanford, CA 94350

**Keywords:** SARS-CoV-2, Nsp1, ribosome-associated quality control, ribosome collision, Alzheimer’s disease

## Abstract

An overarching goal of aging and age-related neurodegenerative disease research is to discover effective therapeutic strategies applicable to a broad spectrum of neurodegenerative diseases. Little is known about the extent to which targetable pathogenic mechanisms are shared among these seemingly diverse diseases. Translational control is critical for maintaining proteostasis during aging. Gaining control of the translation machinery is also crucial in the battle between viruses and their hosts. Severe acute respiratory syndrome coronavirus 2 (SARS-CoV-2) is the causative agent of the ongoing COVID-19 pandemic. Here, we show that overexpression of SARS-CoV-2–encoded nonstructural protein 1 (Nsp1) robustly rescued neuromuscular degeneration and behavioral phenotypes in *Drosophila* models of Alzheimer’s disease, Parkinson’s disease, and amyotrophic lateral sclerosis. These diseases share a common mechanism: the accumulation of aberrant protein species due to the stalling and collision of translating ribosomes, leading to proteostasis failure. Our genetic and biochemical analyses revealed that Nsp1 acted in a multipronged manner to resolve collided ribosomes, abort stalled translation, and remove faulty translation products causative of disease in these models, at least in part through the ribosome recycling factor ABCE1, ribosome-associated quality-control factors, autophagy, and AKT signaling. Nsp1 exhibited exquisite specificity in its action, as it did not modify other neurodegenerative conditions not known to be associated with ribosome stalling. These findings uncover a previously unrecognized mechanism of Nsp1 in manipulating host translation, which can be leveraged for combating age-related neurodegenerative diseases that are affecting millions of people worldwide and currently without effective treatment.

## Significance

Age-related neurodegenerative disease is a looming public health crisis. Despite intensive efforts, effective disease-modifying treatment is still elusive. Development of effective therapeutic strategies applicable to a broad spectrum of neurodegenerative diseases is desirable, but little is known about the extent to which pathogenic mechanisms are shared among these diseases. We discovered that Nsp1, a viral protein encoded by SARS-CoV-2, the virus causing the ongoing COVID-19 epidemic, ameliorates neuromuscular degeneration in fly models of Alzheimer’s disease, Parkinson’s disease, and amyotrophic lateral sclerosis, which share a common mechanism—accumulation of aberrant protein species due to stalling and collision of ribosomes—which is resolved by Nsp1. These results have important implications for the understanding and treatment of neurodegenerative diseases as well as COVID-19.

Proteostasis refers to a cellular state in which protein synthesis, folding, and degradation are maintained at a homeostatic state, such that an intact proteome is preserved (1). Cellular capacity to preserve proteostasis declines with age, which is assumed to contribute to the pathogenesis of age-related diseases (2). Proteostasis failure manifested as formation of aberrant protein aggregates is a defining feature of neurodegenerative diseases (3). The root cause of the proteostasis failure and protein aggregation is still enigmatic. Problems of proteostasis can begin with nascent peptide chains (NPCs) still associated with translating ribosomes, necessitating the deployment of ribosome-associated quality control (RQC) to handle faulty translation (4). During translation elongation, ribosome slowdown and stalling can occur for various reasons. Some are functional and serve to facilitate cellular dynamics. Others are detrimental and can be triggered by damaged mRNAs, mRNA secondary structures, insufficient supply of aminoacyl-tRNAs, or environmental stress (5, 6). Ribosome slowdown and stalling can result in ribosome collision (7), which is sensed by cells as a proxy for aberrant translation and triggers RQC (4, 8–11). Key factors involved in the process are the ubiquitin ligase ZNF598 and the 40S subunit protein Rack1, which recognize the distinct 40S–40S interface of collided ribosomes and promote ubiquitination of specific 40S proteins (12, 13), and the ASC complex that disassembles the leading collided ribosome (14, 15). This then triggers a series of downstream events, including ribosome subunit splitting and recycling by ABCE1 (16), CAT-tailing modification of NPCs still attached to the 60S subunit (17), and degradation of stalled NPCs. The importance of this ribosome-mediated QC process is highlighted by the findings that RQC factors regulating translation elongation and termination are critical for neuronal function and integrity (18–20), and that inefficient RQC results in translation stalling and accumulation of faulty translation products that perturb proteostasis and contribute to Alzheimer’s disease (AD) (21), Parkinson’s disease (PD) (22), and amyotrophic lateral sclerosis (ALS) (23).

Translational control is also crucial in the battle between viruses and their hosts during infection. Due to the lack of their own translational apparatus, viruses rely on the protein synthesis machinery of the host cells to complete their life cycle (24). Viral replication requires sustained high levels of active ribosomes to carry out rapid translation of their mRNAs. To achieve this, viruses must manage to neutralize host innate immune defenses and stress responses, which often target the ribosomes and the translation machinery (25). Gaining control of the ribosomes is therefore crucial in the arms race between viruses and their host cells. Of the three stages of translation—initiation, elongation, and termination—much effort has been directed toward control of initiation, the rate-limiting step of translation (24, 25). However, rapid translation of viral RNAs during replication, and certain features of the viral RNA, such as complex secondary structures throughout the genome, may engender translational stress beyond the initiation step that necessitates the deployment of additional regulatory mechanisms.

Severe acute respiratory syndrome coronavirus 2 (SARS-CoV-2) is the causative agent of the ongoing COVID-19 pandemic that has led to a dramatic loss of human life and disruption of social and economic activities worldwide (26). COVID-19 is characterized by a wide spectrum of symptoms, from fever and cough to multiorgan failures (27). The coronavirus family includes SARS-CoV (28), SARS-CoV-2 (26), and Middle-East respiratory syndrome coronavirus (MERS-CoV) (29). SARS-CoV-2 encodes the spike (S), envelope (E), membrane (M), nucleocapsid (N), nonstructural (NSP1-16), and accessory (ORF3a, -3b, -6, -7a, -7b, -8, -9b, -9c, -10, and -14) proteins (30). NSP1 is one of the first SARS-CoV proteins synthesized upon cell entry and deemed a major virulence factor (31). Structural, biochemical, and cell culture studies have implicated NSP1 in inhibiting the translation initiation of host genes through blocking the mRNA entry channel of the 40S ribosome (32–35), and by promoting mRNA degradation (32, 36, 37). NSP1 has also been implicated in viral evasion of host innate immune response (38–40), although the underlying mechanism is unclear. Till now functional studies on NSP1 are performed in vitro or in cell culture. The in vivo effects of Nsp1 remain untested. Recent proteomics studies of COVID-19 autopsies revealed diverse up- and down-regulation of the proteome with limited overlap across tissues (41), emphasizing the need to study tissue-specific effects of viral proteins including Nsp1.

Although rodent and primate models have been developed, their general use for studying the function of individual SARS-CoV-2 viral proteins has been limited by low availability and requirement for special biocontainment facilities. *Drosophila* offers an alternative model system. In addition to its small size, short life cycle, low maintenance cost, and powerful genetics, *Drosophila* shares conserved innate immune responses and other processes commonly hijacked by pathogens (42). Here we describe our systematic investigation into the in vivo effects of SARS-CoV-2–encoded viral proteins in *Drosophila* neuromuscular tissues under normal and disease conditions. This led to an unexpected finding of amelioration of neuromuscular degeneration by Nsp1 in multiple neurodegenerative disease models and new insights into the biochemical function of Nsp1 in manipulating the host translation machinery.

## Results

### The AD-Related Proteostasis Failure and Neuromuscular Degeneration in Amyloid Precursor Protein C-Terminal Fragment (APP.C99) Transgenic Flies Are Rescued by Nsp1.

**Fig. 1.**
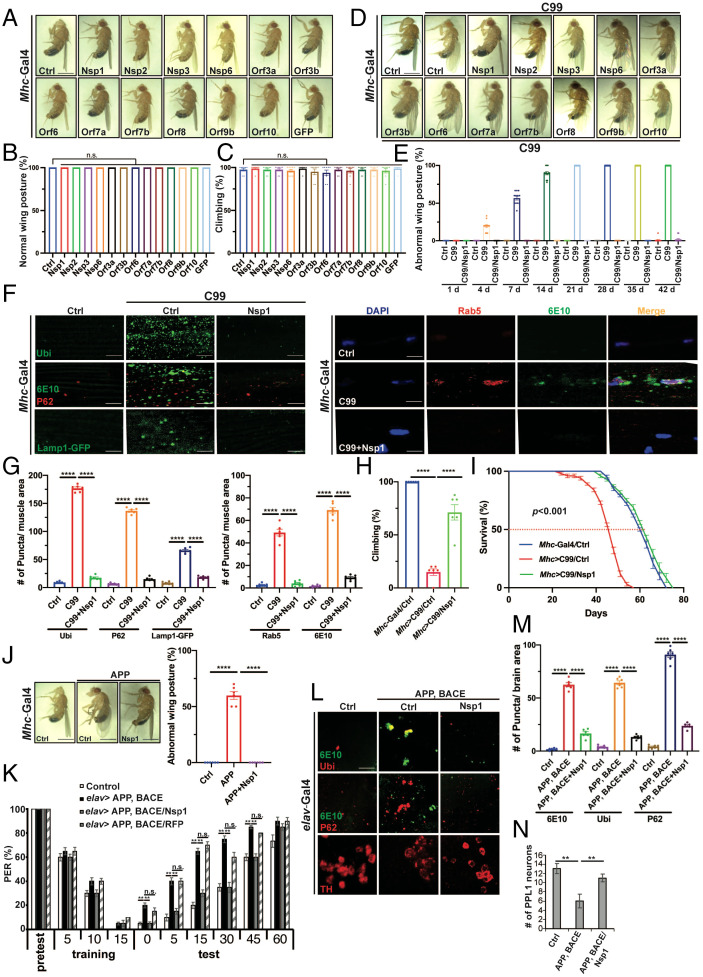
Nsp1 rescues APP/APP.C99-induced neuromuscular defects and memory deficit in *Drosophila.* (*A* and *B*) Images showing effect of muscle expression of SARS-CoV-2–encoded viral proteins on wing posture in wild-type condition. (Scale bar, 1 mm.) Bar graph shows quantification of wing posture defect (*n* = 8 per group) (*B*). (*C*) Quantification of effect of viral protein expression on locomotion (*n* = 8 per group). (*D*) Images showing effect of muscle expression of SARS-CoV-2-encoded viral proteins on wing posture in *Mhc > APP.C99* flies. (Scale bar, 1 mm.) (*E*) Quantification showing effect of Nsp1 on wing posture in *Mhc > APP.C99* flies at different ages (*n* = 12 per group). (*F* and *G*) Immunofluorescent images (*F*) and quantification (*G*) showing effect of Nsp1 on protein aggregation (Ub, p62, and 6E10 staining), lysosomes (Lamp1-GFP), and early endosomes (Rab5) in *Mhc > APP.C99* fly muscle (*n* = 6 per group). (Scale bar, 20 µm in *F*.) (*H* and *I*) Effect of Nsp1 on locomotion (*H*, *n* = 6) and lifespan (*I*) of *Mhc > APP.C99* flies. (*J*) Effect of Nsp1 on wing posture of *Mhc > FL-APP* flies (*n* = 6 per group). (*K*) Effect of Nsp1 on learning and memory in the aversive taste memory assay in *elav > FL-APP/BACE* flies (*n* = 15 per group). (*L*–*N*) Effect of Nsp1 on proteostasis (*L* and *M*) and DA neuron number in the PPL1 cluster (*N*) in *elav > FL-APP/BACE* flies (*n* = 6 per group). 6E10 antibody in *F* and *L* recognizes the very N terminus of APP.C99. (Scale bar, 8 µm in *L*.) Error bars, ± SEM; *P < 0.05, **P < 0.01, ***P < 0.001, ****P < 0.0001, n.s: nonsignificant, in Student’s *t* tests and one-way ANOVA tests.

Although SARS-CoV-2 causes severe respiratory disease, it also affects other organ systems, including the musculoskeletal system (43). To test the potential effect of individual viral proteins on muscle function, we tissue-specifically expressed viral proteins that are likely to engage in intracellular virus–host interactions, using the *Mhc-Gal4* driver and the *UAS-Gal4* system. Twelve SARS-CoV-2 proteins—Nsp1, Nsp2, Nsp3, Nsp6, Orf3a, Orf3b, Orf6, Orf7a, Orf7b, Orf8, Orf9b, and Orf10—were individually expressed. Using two assays—wing posture (Fig. 1 *A* and *B*), a reflection of indirect flight muscle integrity (44), and locomotor activity (Fig. 1*C*), a measure of muscle function—we did not find obvious effect of the individual viral proteins.

We next tested whether the expression of SARS-CoV-2 viral proteins might exacerbate already compromised neuromuscular function in disease conditions. APP.C99 corresponds to the C-terminal fragment of APP resulting from β-secretase cleavage. APP.C99 and its aberrant translation products are emerging as key players in AD pathogenesis (21, 45). Muscle can be a useful system for studying APP-induced toxicity, as APP-derived amyloid pathology in muscle similar to that seen in AD brain is associated with inclusion body myositis (46), a muscle disease, and APP.C99 transgenic mice have been used to model inclusion body myositis (47). As previously reported (21), expression of APP.C99 in the fly muscle resulted in indirect flight muscle degeneration manifested as abnormal held-up or droopy wing postures (*SI Appendix*, Fig. S1*A*). Surprisingly, whereas coexpression of most of the SARS-CoV-2 viral proteins tested had no effect on APP.C99-induced wing posture defect, the coexpression of Nsp1 completely suppressed such defect at different ages (Fig. 1 *D* and *E* and *SI Appendix*, Fig. S1*B*). Expression of Nsp1 protein was confirmed by Western blot and immunostaining (*SI Appendix*, Fig. S1 *C* and *D*). The wing posture defect is known to correlate with APP.C99-induced proteostasis failure manifested as the formation of protein aggregates immunopositive for APP.C99 (detected with 6E10 antibody), ubiquitin (Ubi), and the autophagy receptor p62, and endolysosomal defects, such as enlargement of Rab5^+^ early endosome and accumulation of enlarged lysosomes detected with Lamp1-GFP that are likely caused by altered autophagy flux (21).

Similar APP.C99-inducd endolysosomal defects are observed in mammalian AD models (48–52). APP.C99-induced proteostasis failure (Fig. 1 *F* and *G*) and endolysosomal defects (Fig. 1 *F* and *G*) were effectively suppressed by Nsp1. Muscle expression of APP.C99 also resulted in locomotor defects (Fig. 1*H*) and significantly shortened lifespan (Fig. 1*I*), which were also effectively suppressed by Nsp1. Neuronal expression of APP.C99 using the pan-neuronal *elav-Gal4* driver or the mushroom body-specific *R13F02-Gal4* driver caused proteostasis failure, locomotor and learning and memory deficits, which were also effectively rescued by Nsp1 coexpression (*SI Appendix*, Fig. S1 *E–I*). These results were totally unexpected, as we had initially expected to see exacerbation of the preexisting neuromuscular defects in the APP.C99 model by SARS-CoV-2 viral proteins, considering that postinfection patients with the long-COVID syndrome presented neuromuscular deficits that became persistent with time and caused disabilities, and AD patients were reported to be more susceptible to COVID-19, albeit with undefined mechanism (53).

### The Proteostasis Failure, Neuromuscular Degeneration, and Cognitive Deficits in Full-Length APP-Based Models Are Rescued by Nsp1.

We next tested the effect of Nsp1 on AD-related phenotypes in full-length APP (FL-APP) contexts. Overexpression of FL-APP in the muscle caused wing posture defect, although the penetrance was not as high as in the APP.C99 model, presumably due to the lower level of APP.C99 produced from FL-APP. This phenotype was completely rescued by Nsp1 (Fig. 1*J*).

We also tested the effect of Nsp1 on APP/APP.C99-induced defects in learning and memory. We used transgenic flies coexpressing FL-APP and β-secretase (BACE1) in neurons under *elav-Gal4* control for the behavioral assay. BACE1 was included to facilitate APP.C99 production from FL-APP. We measured aversive taste memory controlled by a neural circuit involving dopaminergic input to neurons in mushroom body—the memory center of the flies—and mushroom body output neurons directing taste response (54). Applying sucrose solution (appetitive tastant) to the tarsi (feet) of a starved fly induced robust feeding behavior as measured by the proboscis extension reflex (PER). After rounds of paired application of sucrose to the tarsi and quinine (aversive tastant) to the proboscis, normal flies learned from experience and showed attenuated PER response to subsequent application of sucrose alone. Compared to control flies, *elav-Gal4 > FL-APP/BACE* flies presented impaired aversive taste memory as indicated by the higher PER response (Fig. 1*K*). Importantly, Nsp1 coexpression effectively attenuated the PER response in *elav > FL-APP/BACE* flies, whereas *UAS-RFP* control had no effect (Fig. 1*K*). Nsp1 coexpression also rescued the proteostasis defect in *elav > FL-APP/BACE* fly brain, as shown by the removal of ubiquitin^+^ and p62^+^ protein aggregates (Fig. 1 *L* and *M*). The *elav > FL-APP/BACE* flies present neurodegeneration phenotypes as shown by the loss of dopaminergic neurons in the PPL1 cluster. This phenotype was also rescued by Nsp1 (Fig. 1*N*). Muscle expression of FL-APP/BACE in *Mhc > FL-APP/BACE* flies also caused proteostasis failure manifested as the formation of protein aggregates immunopositive for ubiquitin and p62 (*SI Appendix*, Fig. S1*J*) and abnormal wing posture (*SI Appendix*, Fig. S1*K*), which were both suppressed by Nsp1 (*SI Appendix*, Fig. S1 *J* and *K*). These data demonstrate that Nsp1 potently rescues AD-related key pathological features induced by FL-APP and FL-APP-derived APP.C99.

### Nsp1 Promotes Removal of Aberrant APP.C99 Species Resulting from Inadequate RQC of Ribosome Stalling.

**Fig. 2.**
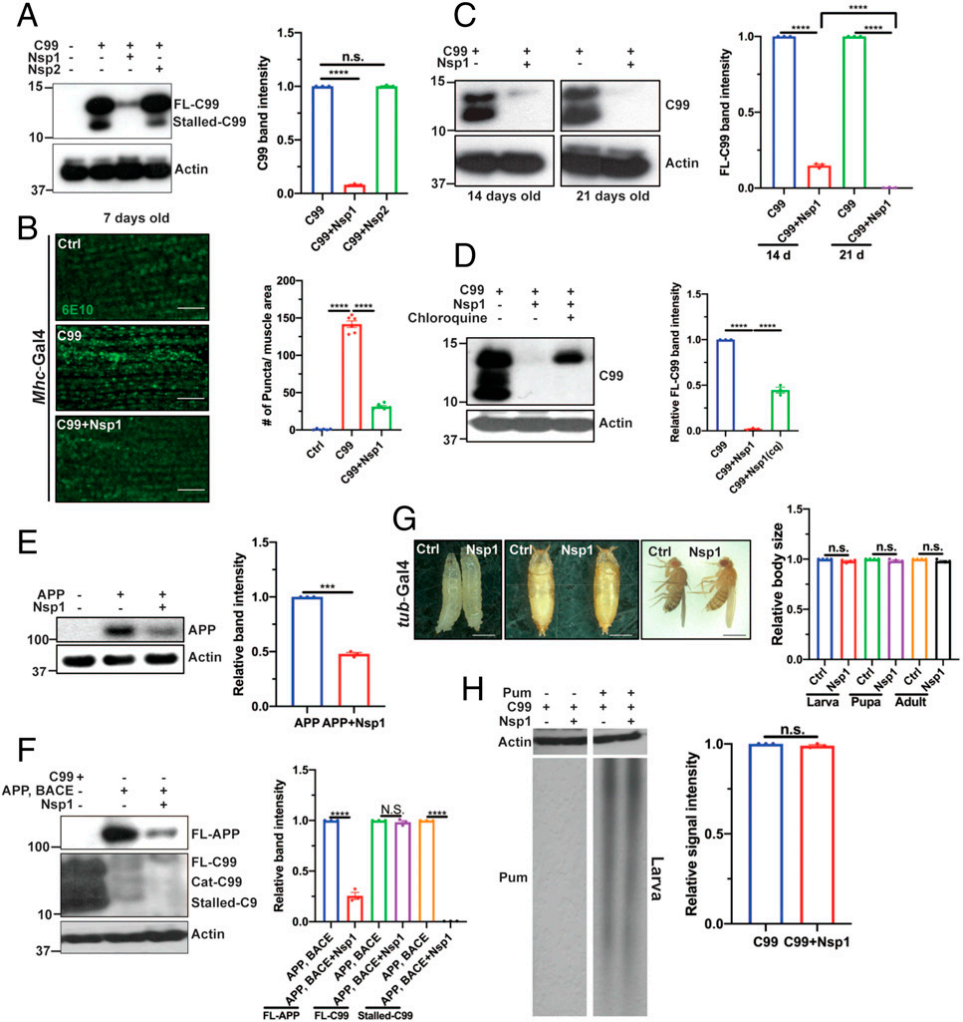
Nsp1 regulates APP/APP.C99 protein expression. (*A*) Immunoblots and quantification (*n* = 3) showing effect of Nsp1, and Nsp2 on FL-APP.C99 (*Upper* band) and internally stalled APP.C99 (*Lower* band) levels. (*B*) Immunostaining and quantification (*n* = 6) showing effect of Nsp1 on APP.C99 expression in *Mhc > APP.C99* fly muscle. (Scale bar, 20 µm.) (*C*) Immunoblots and quantification (*n* = 3) showing effect of Nsp1 on APP.C99 protein level in *Mhc > APP.C99* flies at different ages. (*D*) Immunoblots and quantification (*n* = 3) showing effect of chloroquine treatment on APP.C99 level in *Mhc > APP.C99* flies coexpressing Nsp1. (*E*) Immunoblots and quantification (*n* = 3) showing effect of Nsp1 on FL-APP level in *Mhc > APP* flies. (*F*) Immunoblots and quantification (*n* = 3) showing effect of Nsp1 on FL-APP and APP.C99 levels in brain tissues of *elav > FL-APP/BACE* flies. Cat-C99: CAT-tailed C99. (*G*) Images and quantification (*n* = 6) comparing body sizes of control and *tubulin-Gal4 > Nsp1* flies at the larvae, pupae, and adult stages. (Scale bars, 1 mm.) (*H*) Immunoblots and quantification (*n* = 3) showing Pum labeling of newly synthesized peptides in *Mhc > APP.C99* flies with or without Nsp1 coexpression. Error bars, ± SEM; ***P < 0.001, ****P < 0.0001, n.s: nonsignificant, in Student’s *t* tests and one-way ANOVA tests.

We next investigated the molecular mechanism by which Nsp1 rescues APP.C99-induced pathologies. It was recently shown that during the cotranslational translocation of APP and APP.C99, ribosomes stall on the endoplasmic reticulum (ER) translocon at two locations, one at the stop codon site and another at an internal site ∼30 to 40 aa upstream of the stop codon site (21). Stalling at the stop codon site was associated with decreased level of the ribosome splitting and recycling factor ABCE1, whereas the internal stalling might be caused by ER-targeting, translocon-gating, and cotranslational protein folding and membrane insertion (21), events intrinsic to APP.C99 biogenesis and membrane topogenesis that are likely to slow down translation and cause ribosome collision. In *Mhc > APP.C99* flies coexpressing Nsp1, but not Nsp2, internally stalled APP.C99 was virtually undetectable by Western blot in newly eclosed flies (Fig. 2*A*). Only upon longer exposure was a faint signal detected (*SI Appendix*, Fig. S2*A*). That the lower band corresponded to internally stalled APP.C99 was based on its differential reactions to antibodies against the very N terminus (6E10) or the very C terminus (C.1/6.1) of APP.C99. Whereas stop codon-stalled FL-APP.C99 reacted with both 6E10 and C.1/6.1, internally stalled APP.C99 only reacted with 6E10 (*SI Appendix*, Fig. S2*B*). FL-APP.C99 level was also significantly reduced in Nsp1 coexpressed flies compared to flies without Nsp1 coexpression, consistent with immunostaining for APP.C99 (Fig. 2*B*). We interpret the remaining FL-APP.C99 protein in Nsp1 coexpressing flies as those APP.C99 proteins that have successfully passed the stall, completed the cotranslational translocation, and entered the ER lumen. As flies aged, the level of FL-APP.C99 species also gradually diminished (Fig. 2*C*), suggesting that Nsp1 might act through yet another mechanism to down-regulate APP.C99 protein level, for example by inhibition of new round of translation or promoting protein turnover. Treatment with chloroquine, which blocks the binding of autophagosomes to lysosomes, partially blocked the Nsp1 effect on APP.C99 protein abundance (Fig. 2*D*), supporting the involvement of autophagy/lysosomes.

We also examined the effect of Nsp1 on FL-APP. The level of FL-APP was significantly reduced in *Mhc > FL-APP* (Fig. 2*E*) and *Mhc > FL-APP/BACE* muscle tissues expressing Nsp1 (*SI Appendix*, Fig. S2*C*). Moreover, in *Mhc > FL-APP/BACE* (*SI Appendix*, Fig. S2*C*) and *Mhc > FL-APP* (*SI Appendix*, Fig. S2*D*) flies both the internally stalled and stop codon-stalled FL-APP.C99 species were effectively removed by Nsp1. In the brain tissues of *elav > FL-APP/BACE* flies coexpressing Nsp1, internally stalled APP.C99 was dramatically reduced, whereas FL-APP.C99 was less affected (Fig. 2*F*), suggesting that FL-APP.C99 level might be differentially regulated by Nsp1 in different cell types. FL-APP level was also significantly reduced by Nsp1 in *elav > FL-APP/BACE* fly brain (Fig. 2*F*). Moreover, a dose-dependent effect of Nsp1 on APP.C99 level was observed when one copy or two copies of the *UAS-Nsp1* transgene were coexpressed with APP.C99 in photoreceptor neurons using the *GMR-Gal4* driver (*SI Appendix*, Fig. S2*E*). Nsp1 thus rescues APP.C99-associated toxicity by removing translationally stalled APP/APP.C99 species and downregulating overall APP/APP.C99 levels.

### Nsp1 Does Not Cause Global Shutdown of Protein Synthesis or Turnover of *APP.C99* mRNA in *Drosophila*.

Nsp1 is a virulence factor proposed to restrict cellular gene expression by inhibiting translation through blocking the mRNA entry channel of the 40S ribosomal subunit and by promoting global mRNA degradation (31). Whether Nsp1 affects host mRNA translation or degradation at an organismal level has not been examined. Muscle, neuronal, or body-wide expression of Nsp1 alone had no obvious detrimental effect in flies. Ubiquitous Nsp1 also had no effect on body size (Fig. 2*G*), a sensitive readout of global translation activity in flies as shown by the “minute” phenotype caused by inhibition of translation (55). We suspected that Nsp1 might not act as a strong repressor of global translation, at least in *Drosophila* tissues. This was supported by the relatively unchanged expression levels of actin and other proteins we checked. If anything, at least in the case of ABCE1 its level might be modestly increased by Nsp1 (*SI Appendix*, Fig. S2*F*). To further test if Nsp1 affects global translation in *Drosophila* tissues, we used a new approach to measure protein synthesis in *Drosophila* tissues, which is based on the incorporation of puromycin (Pum) into NPCs. Pum-labeled newly synthesized peptides were then detected using an anti-Pum antibody (56). With this method, we did not find significant difference in new protein synthesis in the muscle tissues of animals with or without Nsp1 coexpression (Fig. 2*H*). We also performed qRT-PCR to analyze mRNA level and did not find evidence of degradation of *APP.C99* mRNA (*SI Appendix*, Fig. S2*G*). The mRNA levels of several other host genes we tested were either not affected or increased (*SI Appendix*, Fig. S2*G*). Although further proteomics and transcriptomics studies are needed to assess Nsp1 effect on host protein and mRNA expression, our data indicate that the effect of Nsp1 on APP/APP.C99 expression is unlikely through mRNA degradation or inhibition of global translation.

### Nsp1 Aborts Stalled Translation.

**Fig. 3.**
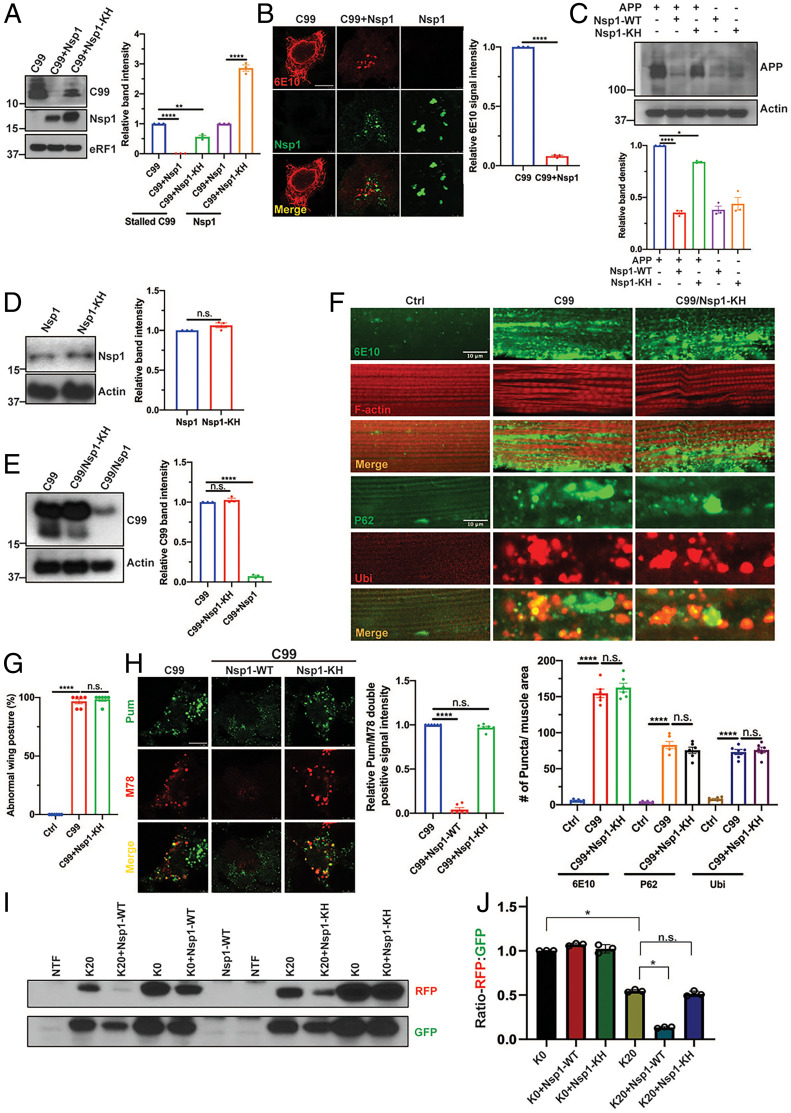
Nsp1 aborts stalled translation. (*A*) Immunoblots and quantification (*n* = 3) showing effect of Nsp1 and Nsp1-KH mutant on APP.C99 expression in HeLa cells. eRF1 serves as loading control. (*B*) Immunostaining and quantification (*n* = 3) showing effect of Nsp1 on APP.C99 protein expression in HeLa cells. (Scale bars, 12 µm.) (*C*) Immunoblots and quantification (*n* = 3) showing effect of Nsp1 and Nsp1-KH on FL-APP expression. The weak signal in cells not transfected with APP cDNA likely represents endogenous APP. (*D*) Immunoblots and quantification (*n* = 3) showing expression levels of Nsp1 and Nsp1-KH in Mhc-Gal4 driven transgenic flies. (*E*) Immunoblots and quantification (*n* = 3) showing effect of Nsp1 and Nsp1-KH on APP.C99 expression in *Mhc > APP.C99* flies. (*F*) Immunostaining and quantification (*n* = 6) showing effect of Nsp1-KH on APP.C99-induced proteostasis failure in *Mhc > APP.C99* flies. (Scale bar, 10 µm.) (*G*) Quantification of effect of Nsp1-KH on APP.C99-induced wing posture defect in *Mhc > APP.C99* flies (*n* = 6). (*H*) Immunostaining and quantification (*n* = 3) of HeLa cells cotransfected with APP.C99 and Nsp1 or Nsp1-KH with the amyloid conformation-specific mOC78 (M78) antibody and anti-Pum, which labeled stalled NPCs after active ribosomes were let run off by HHT treatment. (Scale bar, 8 µm.) (*I* and *J*) Immunoblots (*I*) and data quantification (*J, n* = 3) showing effect of Nsp1-WT and Nsp1-KH mutant on RFP and GFP expression from the stall reporter GFP-P2A-K20-P2A-RFP and control reporter GFP-P2A-K0-RFP. *P < 0.05, **P < 0.01, ***P < 0.001, ****P < 0.0001, n.s: nonsignificant, in Student’s *t* tests and one-way ANOVA tests.

Translation stalling of APP.C99 also occurs in mammalian cells (21). As in fly tissues, Nsp1 dramatically lowered stalled translation products of APP.C99 (Fig. 3*A*) and overall APP.C99 expression as detected by immunostaining (Fig. 3*B*) in HeLa cells. In contrast, an Nsp1 mutant containing the K164A/H165A (Nsp1-KH) mutations, which abolish the ribosome-binding of Nsp1 (33), was less effective in reducing stalled translation products of APP.C99, although itself was expressed at a higher level than wild-type Nsp1 (Fig. 3*A*). Nsp1, but not Nsp1-KH, also significantly reduced FL-APP (Fig. 3*C*). To test the in vivo effect of Nsp1-KH, we made *UAS-Nsp1-KH* transgenic flies. When expressed at comparable level as wild-type Nsp1 (Fig. 3*D*), Nsp1-KH had no obvious effect on stalled APP.C99 translation (Fig. 3*E*), nor APP.C99-induced proteostasis failure (Fig. 3*F*) and wing posture defect (Fig. 3*G*). We next used mammalian cells to investigate the mechanism of Nsp1 regulation of stalled translation. First, we performed Pum labeling of stalled NPCs. This involved pretreatment of cells with homoharringtonine (HHT), which allows elongating/active ribosomes to run off but prevents new rounds of translation (57). This was followed by a combined emetine and Pum treatment to incorporate tRNA-like Pum to the C termini of ribosome-stalled NPCs (57). This way, Nsp1 but not Nsp1-KH was found to significantly reduce the level of stalled NPCs in APP.C99-expressing cells (Fig. 3*H*).

Next, we used the GFP-P2A-K20-P2A-RFP reporter to assess the effect of Nsp1 on ribosome stalling. In this reporter, the GFP, K20, and RFP reporters are used to monitor overall mRNA translation, translational stalling by 20 consecutive K residues, and read-through of the stall site, respectively, with the self-cleaving P2A allowing each reporter to be independent marker of translation. As a control, we used the GFP-P2A-K0-RFP reporter without a stall signal. In contrast to situations in which the early RQC pathway was disabled, as in ZNF598 knockdown condition, that resulted in read-through of the K20 stall and increased RFP/GFP ratio (12), Nsp1 cotransfection significantly reduced the ratio of RFP/GFP expressed from the GFP-P2A-K20-P2A-RFP reporter compared to without Nsp1 (Fig. 3 *I* and *J*), suggesting that read-through of the K20 stall was further blocked. The Nsp1-KH mutant was less effective in this assay (Fig. 3 *I* and *J*). No effect on the ratio of RFP/GFP expressed from the nonstalling GFP-P2A-K0-RFP reporter was observed for Nsp1 or Nsp1-KH (Fig. 3 *I* and *J*), although Nsp1 apparently inhibited overall translation of both reporters in mammalian cells.

Using another translation stall reporter, GFP-P2A-Flag-K20-P2A-mKate2, in which the arrested translation product can be detected with the Flag antibody, we found that Nsp1 reduced the mKate2/GFP ratio, consistent with the GFP-P2A-K20-P2A-RFP reporter data, and levels of both FL-Flag-K20 and arrested Flag-K20 products, suggesting that it aborted stalled translation (*SI Appendix*, Fig. S3*A*). Importantly, the effect of Nsp1 in aborting stalled translation was dependent on early RQC factors—the ribosome collision sensor ZNF598 and the ribosome disassembly factor ASCC3—as FL-Flag-K20 level and mKate2/GFP ratio were both increased when ZNF598 or ASCC3 was inhibited, irrespective of Nsp1 presence (*SI Appendix*, Fig. S3 *B* and *C*). Together with the results of Pum labeling of stalled NPCs, these data support the hypothesis that a normal activity of Nsp1 is to abort stalled translation. In SARS CoV-2 infection condition, this presumably serves to promote the disassembly of stalled ribosomes on viral RNAs to prevent the accumulation of aberrant viral proteins, or to recycle ribosomes stalled on host mRNAs to make them available for viral translation. The reduction of total FL-APP and APP.C99 levels by Nsp1 observed earlier is also consistent with this abortive termination mode of NSP1 action on stalled ribosomes.

### Nsp1 Promotes Resolution of Collided Ribosomes and Inhibits cGAS/STING Signaling.

**Fig. 4.**
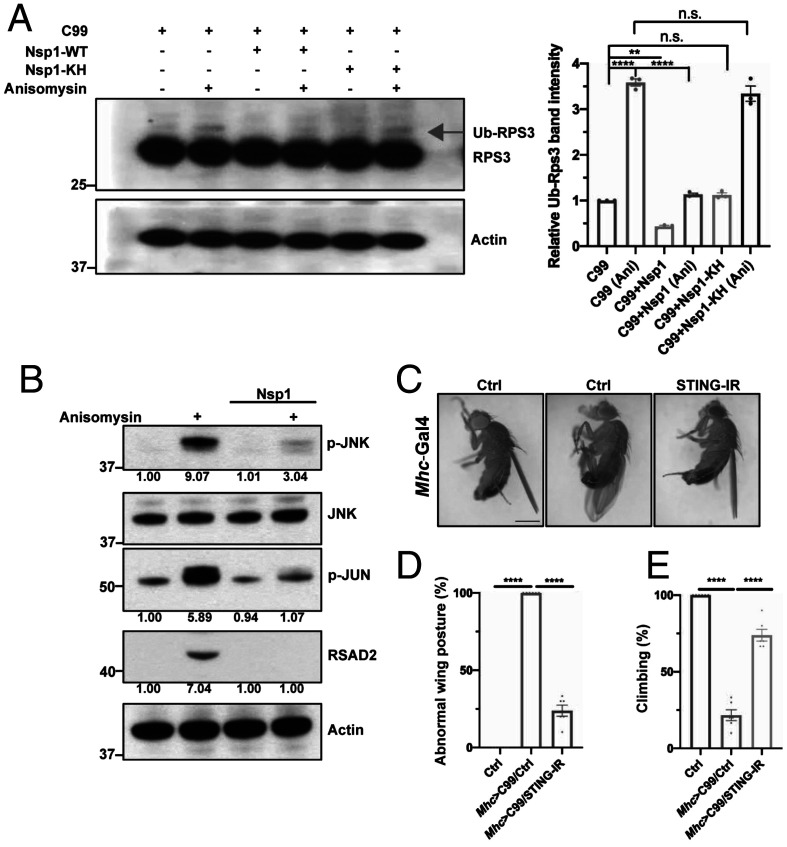
Nsp1 resolves collided ribosomes and down-regulates cGAS/STING signaling. (*A*) Immunoblots and quantification (*n* = 3) showing effect of Nsp1 and Nsp1-KH on ribosome collision-induced Rps3 ubiquitination in APP.C99 transfected HeLa cell with or without Ans treatment. Arrow marks ubiquitinated Rps3. (*B*) Immunoblots showing effect of Nsp1 on innate immune signaling (p-JNK, p-Jun) and interferon stimulated gene (RSAD2) expression promoted by Ans-induced ribosome collision. Data represents two independent experiments. (*C*–*E*) Images and data quantification (*n* = 6) showing effect of STING RNAi on the wing posture (*C* and *D*) and locomotion (*E*) in *Mhc > APP.C99* flies. Error bars, ± SEM; **P < 0.01, ****P < 0.0001, n.s: nonsignificant, in Student’s *t* tests and one-way ANOVA tests. (Scale bar, 1 mm in *C*.)

To further test the mechanism of Nsp1 action in handling stalled translation, we assessed the effect of Nsp1 on the ubiquitination of 40S subunit Rps3, an indicator of ribosome collision (12, 13). Consistent with APP.C99 translation causing ribosome collision, Rps3 ubiquitination was elevated in APP.C99 transfected HeLa cells. We also used low concentration of anisomycin (Ans) to induce ribosome collision and Rps3 ubiquitination (58). Nsp1 significantly reduced collision-induced Rps3 ubiquitination in APP.C99-expressing and Ans treatment conditions, whereas Nsp1-KH was less able to do so (Fig. 4*A*), consistent with Nsp1 resolving collided ribosomes. To further test this hypothesis, we performed sucrose gradient analysis of ribosomes and examined the distribution of ASCC3 and EDF1, factors preferentially associating with collided ribosomes (14, 59). Nsp1 did not change the distribution of 40S protein Rps3 across the sucrose gradient but reduced the abundance of ASCC3 and EDF1 in the polysome fractions in HeLa cells (*SI Appendix*, Fig. S4*A*). This was also observed for EDF1 in Ans-treated HeLa cells (*SI Appendix*, Fig. S4*A*). Together, these results support the notion that Nsp1 promotes resolution of collided ribosomes.

The cyclic GMP-AMP synthase-stimulator of interferon genes (cGAS/STING) pathway senses cytosolic DNA and induces interferon signaling to activate the innate immune system. Translation stress and collided ribosomes can serve as coactivators of cGAS (60), with ribosome collision leading to cytosolic localization of cGAS, which preferentially interacts with collided ribosomes, and the ribosome association stimulates cGAS activity. Using cytosolic cGAS localization as a proxy of its activation (60), we found that Nsp1 significantly attenuated ribosome collision-induced cGAS cytoplasmic distribution and thus activation (*SI Appendix*, Fig. S4 *B* and *C*). Western blot analysis of subcellular fractionation confirmed the relative nuclear enrichment of cGAS in Nsp1-expressing cells (*SI Appendix*, Fig. S4*D*). These results were correlated with attenuated innate immune signaling as monitored with p-JUN and p-JNK, and expression of the interferon-stimulated gene RSAD2 (Fig. 4*B*). Moreover, the effect of Nsp1 in suppressing the cytosolic distribution of cGAS was at least partially dependent on the early acting RQC factors ZNF598 and ASCC3 that disassemble collided ribosomes (*SI Appendix*, Fig. S4 *B–D*). These data further corroborate the role of Nsp1 in resolving collided ribosomes and offer a mechanism of innate immune evasion by SARS-CoV-2. We also tested if ribosome collision-induced cGAS/STING signaling is relevant to the neuromuscular toxicity of APP.C99. The abnormal wing posture and locomotor defects in *Mhc > APP.C99* flies was effectively rescued by RNAi of the fly homolog of STING (Fig. 4 *C*–*E*), supporting that cGAS/STING signaling contributes to ribosome collision-related neuromuscular toxicity.

### The Ribosome Splitting and Recycling Factor ABCE1 Mediates Nsp1 Effect on Stalled APP.C99 Translation.

**Fig. 5.**
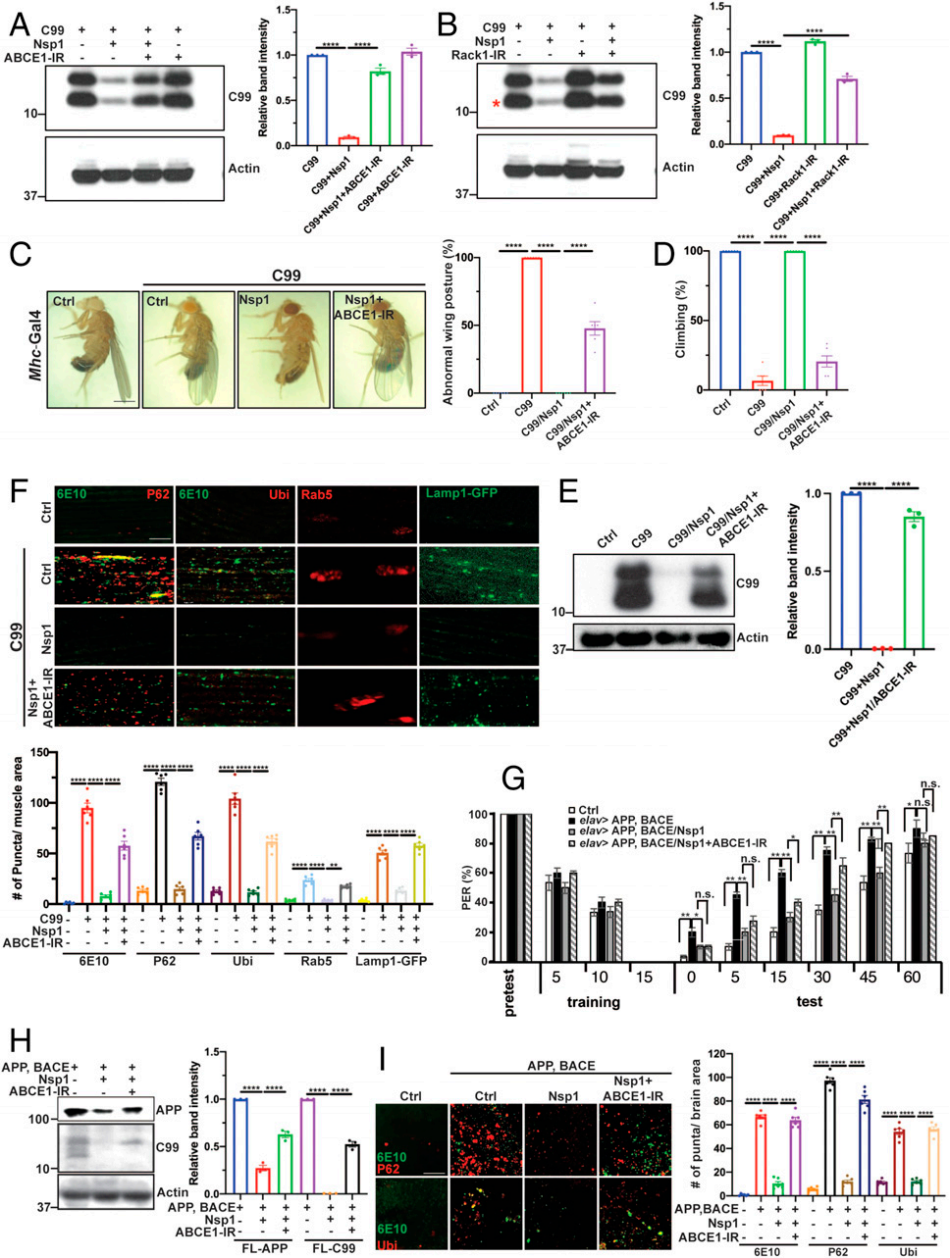
ABCE1 mediates the effect of Nsp1 on APP/APP.C99 translation and toxicity. (*A* and *B*) Immunoblots and quantification (*n* = 3) showing the effect of ABCE1 RNAi (*A*) and Rack1 RNAi (*B*) on Nsp1 inhibition of APP.C99 protein expression in HeLa cells. (*C* and *D*) Images and data quantification (*n* = 6) showing effect of ABCE1 RNAi on the rescue of wing posture (*C*) and locomotion (*D*) defects by Nsp1 in *Mhc > APP.C99* flies. (Scale bar, 1 mm in *C*.) (*E*) Immunoblots and quantification (*n* = 3) showing effect of ABCE RNAi on the inhibition of stalled APP.C99 protein levels by Nsp1. (*F*) Immunostainings and quantification (*n* = 6) showing the effect of ABCE1 RNAi on the rescue of proteostasis failure and endolysosomal defects in *Mhc > APP.C99* flies by Nsp1. (Scale bar, 20 µm.) (*G*) Effect of ABCE1 RNAi on the rescue of aversive taste memory deficit in *elav > FL-APP/BACE* flies by Nsp1 (*n* = 15). (*H*) Immunoblots and quantification (*n* = 3) showing effect of ABCE1 RNAi on the level of FL-APP and APP.C99 removed by Nsp1 in *elav > FL-APP/BACE* fly brains. (*I*) Immunostainings and quantification (*n* = 6) showing effect of ABCE1 RNAi on the level of Ub^+^ and p62^+^ aggregates in *elav > FL-APP/BACE* fly brains. (Scale bar, 8 µm.) Error bars, ± SEM; *P < 0.05, **P < 0.01, ***P < 0.001, ****P < 0.0001, n.s: nonsignificant, in Student’s *t* tests and one-way ANOVA tests.

To understand the mechanism of action of Nsp1 in regulating ribosome stalling, we searched published reports on the Nsp1-interactome for potential interaction between Nsp1 and RQC-related factors. One study reported the presence of ABCE1, together with translation initiation and elongation factors, in the immunocomplex captured with a 3xFlag-Nsp1 construct (33), although the functional significance of these interactions was unknown. Given the critical role of ABCE1 in regulating ribosome splitting and recycling during RQC (22, 61), we tested if ABCE1 is required for Nsp1 to abort stalled APP.C99 translation. ABCE1 knockdown, with the knockdown efficiency confirmed by Western blot (*SI Appendix*, Fig. S5*A*), resulted in recovery of stalled APP.C99 species removed by Nsp1 in HeLa cells (Fig. 5*A*). We also tested other RQC factors for possible role in mediating the effect of Nsp1 on stalled APP.C99 translation. Knockdown of Rack1, a 40S ribosomal protein previously implicated in regulating 40S subunit ubiquitination and resolving stalled ribosomes (13), also resulted in partial recovery of stalled APP.C99 species removed by Nsp1 (Fig. 5*B* and *SI Appendix*, Fig. S5*B*). Intriguingly, although ABCE1 and eRF1 work in a complex to terminate normal translation at stop codons (62), unlike ABCE1, the knockdown of eRF1 had no obvious effect on the removal of stalled APP.C99 by Nsp1 (*SI Appendix*, Fig. S5 *C* and *D*). Furthermore, although the eIF3 complex involved in initiation was shown to associate with Nsp1 directly or indirectly (33, 63), knocking down a key eIF3 component eIF3E had no obvious effect on Nsp1’s ability to abort stalled APP.C99 translation (*SI Appendix*, Fig. S5*E*). These data support a specific role of ABCE1 in mediating the effect of Nsp1 in regulating stalled APP.C99 translation.

### Nsp1 Employs a Multipronged Strategy to Manipulate Stalled APP.C99 Translation in *Drosophila* AD Models.

We further examined the role of ABCE1 in mediating the effect of Nsp1 in suppressing APP.C99-induced neuromuscular pathology in vivo. ABCE1 interfering RNA (RNAi) partially blocked the effect of Nsp1 on APP.C99-induced wing posture (Fig. 5*C*) and locomotion (Fig. 5*D*) defects in an age-dependent manner (*SI Appendix*, Fig. S5*F*), concomitant with ABCE1 RNAi age-dependently recovering the stalled APP.C99 species removed by Nsp1 (Fig. 5*E*). The age-dependent effect was likely due to incomplete knockdown of ABCE1 by the RNAi transgene and age-related decline of ABCE1 level, such that the knockdown of ABCE1 was more complete in older flies. This was confirmed by RT-PCR and Western blot analyses of *ABCE1* RNA and protein levels (*SI Appendix*, Fig. S5 *G* and *H*). As in mammalian cells, eRF1 RNAi was ineffective in blocking the Nsp1 effect (*SI Appendix*, Fig. S5*I*). This lack of effect by eRF1 RNAi ruled out any possible explanation of the ABCE1 effect as caused by the RNAi process or titration of Gal4 by an added copy of *UAS* transgene, therefore supporting the specificity of the ABCE1–Nsp1 interaction. ABCE1 RNAi also blocked the effect of Nsp1 in rescuing the proteostasis failure and endolysosomal defects caused by APP.C99 (Fig. 5*F*). In the aversive taste memory assay, ABCE1 RNAi effectively blocked the Nsp1 effect in rescuing the memory deficit in *elav > FL-APP/BACE* flies (Fig. 5*G*), whereas eRF1 RNAi had no significant effect (*SI Appendix*, Fig. S5*J*). Moreover, the effect of Nsp1 in reducing FL-APP and APP.C99 expression in *elav > FL-APP/BACE* flies was also partially blocked by ABCE1 RNAi (Fig. 5*H*), but not eRF1 RNAi (*SI Appendix*, Fig. S5*K*). Correspondingly, ABCE1 RNAi attenuated the rescuing effect of Nsp1 on proteostasis failure in brain tissues of *elav > FL-APP/BACE* flies (Fig. 5*I*).

Previous studies implicated mechanistic target of rapamycin complex 2 (mTORC2)-AKT signaling in regulating the RQC process (23). We found that knockdown of fly AKT partially recovered FL-APP.C99 species removed by Nsp1, suggesting that AKT signaling also mediates the effect of Nsp1 in the handling of stalled APP.C99 (*SI Appendix*, Fig. S5*L*), although the internally stalled APP.C99 species was not as efficiently recovered as in the ABCE1 RNAi condition. Furthermore, knockdown of ATG1, a key regulator of the autophagy process that was previously implicated in regulating APP.C99 toxicity (21), also partially recovered FL-APP.C99 but not the internally stalled lower APP.C99 species removed by Nsp1 (*SI Appendix*, Fig. S5*L*). Consistent with autophagy playing a key role in regulating aberrant translation products, ATG1 overexpression (OE) efficiently removed internally stalled APP.C99 species (*SI Appendix*, Fig. S5*M*), whereas knockdown of *ATG1* and another autophagy gene *ATG8* resulted in increased levels of internally stalled APP.C99 species (*SI Appendix*, Fig. S5*N*), and enhanced wing posture defect caused by APP.C99 (*SI Appendix*, Fig. S5*O*).

Intriguingly, although ABCE1-RNAi and ATG1 RNAi individually each resulted in recovery of primarily the FL-APP.C99 protein in young flies, their combined RNAi recovered both FL-APP.C99 and internally stalled APP.C99 species (*SI Appendix*, Fig. S5*P*), concomitant with the reappearance of the wing posture defect (*SI Appendix*, Fig. S5*Q*). Moreover, induction of ribosome collision with Ans facilitated the recovery of internally stalled APP.C99 species removed by Nsp1 in ABCE1-RNAi and ATG1 RNAi conditions but had less effect in ABCE1 RNAi/ATG1 RNAi conditions (*SI Appendix*, Fig. S5*R*). Although the biochemical mechanisms by which Nsp1 impinges on ABCE1, AKT, and ATG1 to regulate the abundance of stalled APP.C99 species remain to be elucidated, these results support the notion that Nsp1 deploys a multipronged strategy to handle stalled APP.C99 translation.

### Nsp1 Specifically and Robustly Rescues the Neuromuscular Degeneration Phenotypes in *Drosophila* PD and ALS Models that Also Feature Translation Stalling.

**Fig. 6.**
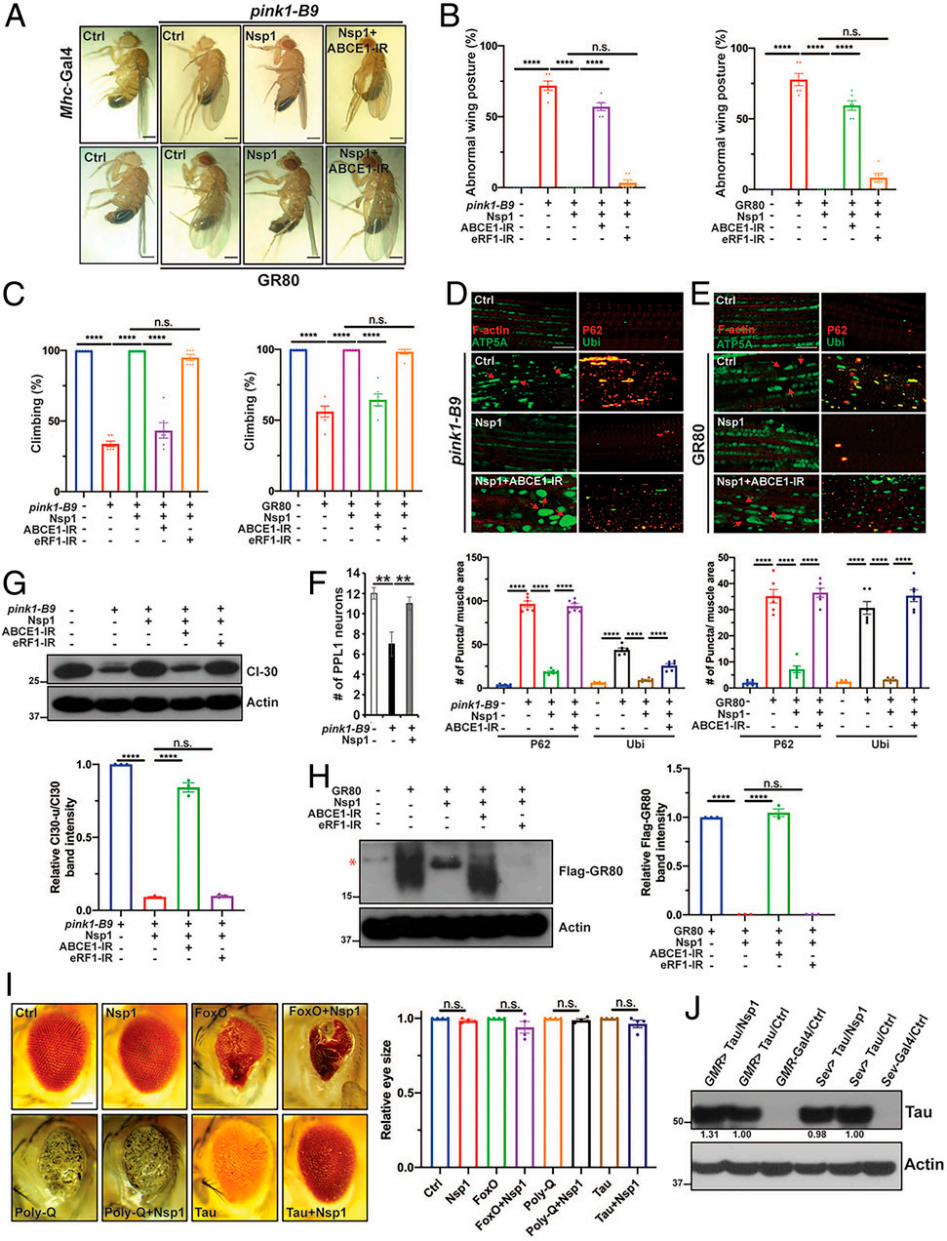
Nsp1 specifically and robustly rescues the neuromuscular degeneration phenotypes in *Drosophila* PD and ALS models. (*A*) Images showing rescue of the abnormal wing posture phenotype of *PINK1^B9^* mutant flies and *Mhc > GR80* flies by Nsp1, and the impact of ABCE1 RNAi on the Nsp1 effect. (Scale bar, 1 mm.) (*B*) Quantification (*n* = 6) of the wing posture phenotype in *PINK1^B9^* mutant flies and *Mhc > GR80* flies after various genetic manipulations. (*C*) Quantification (*n* = 6) of locomotion phenotypes in *PINK1^B9^* mutant flies and *Mhc > GR80* flies after various genetic manipulations. (*D* and *E*) Immunostainings and quantification (*n* = 6) showing effect of Nsp1 on mitochondrial morphology and proteostasis in *PINK1^B9^* mutant flies and *Mhc > GR80* fly muscle. Mitochondria were stained with anti-ATP5A, protein aggregates with anti-p62 and anti-Ub, and muscle fiber with Phalloidin for F-actin. (Scale bars, 20 µm.) (*F*) Quantification showing effect of *TH-Gal4* driven Nsp1 expression on DA neuron number in *PINK1^B9^* mutant flies (*n* = 6). (*G*) Immunoblots and quantification (*n* = 3) showing effect of Nsp1 on stalled translation product of C-I30 in *PINK1^B9^* mutant flies, with or without ABCE1 or eRF1 RNAi. (*H*) Immunoblots and quantification (*n* = 3) showing effect of Nsp1 on stalled GR80 products in *Mhc > GR80* fly muscle, with or without ABCE1 or eRF1 RNAi. The asterisk marks a nonspecific band. (*I*) Images and quantification (*n* = 4) showing effect of Nsp1 coexpression on photoreceptor neuron degeneration caused by dFoxo, polyQ, or tau. (Scale bar, 100 µm.) (*J*) Immunoblots showing effect of Nsp1 on tau protein expression in *GMR > tauV337M* or *sev > tau-WT* flies. Actin serves as loading control. Data represent two independent experiments. Error bars, ± SEM; **P < 0.01, ****P < 0.0001, n.s: nonsignificant, in Student’s *t* tests and one-way ANOVA tests.

Encouraged by the robust effect of Nsp1 in rescuing the disease phenotypes in AD models, we further tested its effect in other disease models. Inefficient resolution of stalled translation also contributes to neuromuscular degeneration in the *PINK1* model of PD (22), and the poly(GR) model of C9ORF72-ALS/frontotemporal degeneration (23). Overexpression of Nsp1 completely rescued the muscle degeneration-induced wing posture defect in *PINK1* mutant flies and *Mhc > GR80* flies expressing 80 GR dipeptide repeats in the fly muscle (Fig. 6 *A* and *B*). This was correlated with rescue of the locomotor deficits in these flies (Fig. 6*C*). At the cellular level, Nsp1 restored proteostasis in the flight muscle as indicated by the significant removal of p62^+^ and Ub^+^ protein aggregates (Fig. 6 *D* and *E*), and it improved mitochondrial morphology (Fig. 6 *D* and *E* and *SI Appendix*, Fig. S6 *A* and *B*). In the *PINK1* model, Nsp1 overexpression also rescued neuron loss (Fig. 6*F*) and restored mitochondrial morphology (*SI Appendix*, Fig. S6 *C* and *D*) in the PPL1 cluster dopaminergic neurons. Importantly, the ability of Nsp1 to rescue the proteostasis failure and mitochondrial morphology defect in both the *PINK1* and *Mhc > GR80* flies was significantly blocked by the knockdown of ABCE1 (Fig. 6 *D* and *E* and *SI Appendix*, Fig. S6 *A* and *B*). The ability of Nsp1 to rescue the wing posture defect caused by muscle degeneration in both the *PINK1* and *Mhc > GR80* flies was also significantly blocked by the knockdown of ABCE1, but not eRF1 (Fig. 6 *B* and *C*).

In *PINK1* mutant flies, inadequate RQC of stalled translation of complex-I 30-kDa subunit (*C-I30*) mRNA at the stop codon site resulted in reduced normal C-I30 protein level and the formation of a CAT-tailed C-I30 species (C-I30-u) (22). Nsp1 efficiently removed C-I30-u and restored normal C-I30 level (Fig. 6*G*), indicating that Nsp1 resolved C-I30 translation stalling. This effect of Nsp1 was significantly attenuated by the knockdown of ABCE1, but not eRF1, demonstrating that ABCE1 specifically mediates the effect of Nsp1 in resolving stalled C-I30 translation (Fig. 6*G*). Similarly, aberrant poly(GR) translation products resulting from inadequate RQC of stalled GR80 translation (23) were also robustly removed by Nsp1, and ABCE1-RNAi, but not eRF1 RNAi, partially blocked this Nsp1 effect (Fig. 6*H*).

We next assessed the specificity of Nsp1 action in rescuing neurodegeneration phenotypes. Photoreceptor neuron degeneration caused by dFoxo overexpression or polyglutamine (polyQ) expansion was not affected by Nsp1 (Fig. 6*I*). Photoreceptor neuron degeneration caused by overexpression of AD-related tau was also not affected by Nsp1 (Fig. 6*I*). At the molecular level, Nsp1 did not affect tau protein expression (Fig. 6*J*). This lack of effect of Nsp1 on tau protein expression or toxicity also argues against its rescue of the neuromuscular degeneration phenotypes in the AD, PD, and ALS models being attributable to a general translational inhibition of disease-associated proteins. Since there is no evidence that the translation of tau is stalled or subjected to RQC (23), these data support that Nsp1 exhibits specificity in its modulation of neuromuscular pathology, preferentially affecting those caused by inadequate RQC of stalled translation.

## Discussion

Our results demonstrate that Nsp1 offers broad neuroprotective effect due to its unique ability to manipulate collided ribosomes and host translation. This finding has far-reaching implications in our understanding and fight against neurodegenerative diseases as well as COVID-19. We show that Nsp1 manipulates the RQC pathway of translational regulation. Our in-depth biochemical and genetic analyses reveal that Nsp1 employs a multipronged strategy to abort stalled translation: disassembly/recycling of collided ribosome, RQC, and autophagy. Nsp1 likely acts through the early RQC factors, which sense ribosome collision, and the ASC complex, which disassembles stalled ribosomes, to abort the translation by ribosomes stalled at the internal sites (*SI Appendix*, Fig. S7). On the other hand, ABCE1 inadequacy has been linked to defective RQC of ribosomes stalled at stop codon sites (22). The fact that in aged flies both the stop codon site stalled and internally stalled APP.C99 species recovered in ABCE1 RNAi condition suggests that either ABCE1 also handles internally stalled ribosomes, which became obvious with more complete ABCE1 knockdown in older flies, or inefficient removal of stalled ribosomes at the stop codon site will gradually lead to collisions with trailing ribosomes to cause internal stalling. Our data also implicate the autophagy pathway in mediating Nsp1 effect on stalled APP.C99 translation. *ATG1* mRNA level was increased in Nsp1 expressing flies (*SI Appendix*, Fig. S2*G*), and ATG1-OE reduced internally stalled APP.C99 species, whereas ATG1 RNAi and ATG8 RNAi had opposite effects (*SI Appendix*, Fig. S5 *M* and *N*). Interestingly, *mTOR* mRNA level was also increased in Nsp1-expressing flies (*SI Appendix*, Fig. S2*G*). Although one of the mTOR signaling complexes, mTORC1, has been established as a negative regulator of autophagy (64), there are mTORC1-independent autophagy pathways (65). Moreover, the other mTOR complex, mTORC2, which activates AKT signaling, has been shown to positively regulate the RQC pathway to remove abnormal translation products (23). Thus, how mTOR, ATG1, and autophagy mediate Nsp1 effect on aberrant translation await further investigation. Finally, the fact that Nsp1 significantly reduces total APP and APP.C99 levels also suggests that it may inhibits new rounds of translation (*SI Appendix*, Fig. S7).

We speculate the following scenarios whereby this recently discovered function of Nsp1 in resolving collided ribosomes may be relevant to its role during natural SARS-CoV-2 infection. 1) It helps recycle ribosomes stalled on host mRNAs to maximize ribosome usage for viral mRNA translation. During rapid viral replication when ribosomes are in high demand, more recycled ribosomes will benefit viral protein synthesis, as SARS-CoV-2 has the ability to shut down new translation of host mRNAs but spare viral mRNAs (34, 66). 2) During rapid translation of viral mRNAs, ribosome collision and stalling are inevitable. Aborting such stalled translation will prevent production of defective viral proteins, which can compromise viral virulence. Recent studies indeed suggest a role of RQC in optimal vaccina virus replication (67, 68). 3) Aborting stalled translation may help SARS-CoV-2 evade host antiviral response. Consistent with collided ribosomes serving as coactivators of cGAS/STING signaling (60), we found that Nsp1 acts through the early RQC factors associated with collided ribosomes to down-regulate cGAS/STING signaling. Thus, by aborting stalled translation and resolving collided ribosomes, Nsp1 may help SARS-CoV-2 evade cGAS/STING-mediated innate immune response.

As one of the first proteins to be synthesized from SARS-CoV-2 genomic RNA after cell entry, Nsp1 is regarded as an important virulence factor thought to manipulate host gene expression by hijacking the translation machinery and shutting off host protein expression. Yet the mechanism of Nsp1 action most critical to viral pathogenesis remains enigmatic. Previous studies in cultured cells indicated that hours after SARS-Cov-2 virus infection, when viral replication had occurred, only small changes of host proteome were discernable (69, 70). Moreover, proteomics studies of COVID-19 autopsies revealed tissue-specific up- and down-regulation of many proteins (41), suggesting that if there is shutdown of host translation by Nsp1, it is selective and not global. This is consistent with our observation of lack of dramatic changes in host protein synthesis in *Drosophila* tissues overexpressing Nsp1.

Age-related neurodegenerative diseases such as AD, PD, and ALS are characterized by the formation of distinct protein aggregates due to proteostasis failure. Although previous studies of proteostasis failure in these diseases have focused on postsynthesis and mature proteins, recent studies indicate that proteostasis failure can originate from NPCs still associated with ribosomes. When RQC activity is inadequate, defective translation products containing CAT-tail–like C-terminal extension will accumulate. In AD, PD, and ALS models, these aberrant protein species perturb proteostasis by forming aggregates and cause endolysosomal, autophagy, and mitochondrial defects (21–23). The cause of RQC impairment in neurodegenerative disease is poorly understood. Previous studies have implicated ABCE1 inadequacy in PD (71) and AD (21) models. ABCE1 is a labile and metastable Fe-S cluster-containing protein whose stability is compromised by mitochondrial stress (22), and whose level declines with age (21), making it a likely contributor to RQC failure in age-related diseases. How other RQC factors may be affected early in the disease process is unknown. As RQC factors are substoichiometric to ribosomes (72), intrinsic and extrinsic stress stimuli that burden the translation machinery will contribute to the deficit of RQC factors needed for handling the translation of problematic transcripts, such as *APP*.

Inefficient resolution of stalled APP/APP.C99 translation may also cause excessive cGAS/STING activation due to persistence of collided ribosomes, leading to neuroinflammation and contributing to AD pathogenesis, as supported by our data. The pathogenic role of cGAS/STING activation in neurodegenerative disease has been implicated by recent studies in animal models of PD (73). Persistent collided ribosomes may also activate stress response pathways that influence cell survival or death decisions (74, 75). By facilitating RQC and resolving stalled translation, Nsp1 coincidentally prevents the formation of aberrant translation products, and the downstream pathophysiology (*SI Appendix*, Fig. S7). Ribosome collision can also trigger *cis*-acting feedback inhibition of translation initiation of problematic mRNAs (59, 76, 77). A potential role of Nsp1 in this process may also be beneficial in the disease settings we tested, as reduced initiation will lower the chance of additional trailing ribosomes to collide with the stalled ribosomes, making it easier for the RQC machinery to handle existing stalled ribosomes (*SI Appendix*, Fig. S7). Inhibition of translation by Nsp1 may also mitigate disease by reducing the burden of misfolded proteins for the already compromised protein QC machinery or by preserving energy resource, as translation is a highly energy-consuming process. Future studies will further test the effects of specifically targeting collided ribosomes in the context of aging and aging-related diseases. In the meantime, our results suggest that therapeutic approaches delivering Nsp1 to disease-relevant cell types may offer a promising strategy to combat these devastating diseases. Implementing these approaches in mammalian systems is one of our future aims.

## Methods

### *Drosophila* Genetics.

Fly culture and crosses were performed according to standard procedures. Adult flies were generally raised at 25 °C and with 12/12-h dark/light cycles. Fly food was prepared with a standard receipt (water, 17 L; agar, 93 g; cornmeal, 1,716 g; brewer’s yeast extract, 310 g; sucrose, 517 g; dextrose, 1,033 g). See *SI Appendix* for sources of the various fly strains used in this study.

### Pum Labeling of Fly Tissue.

Pum labeling of newly synthesized proteins in *Drosophila* larvae was done as described previously (56). Briefly, 5 to 10 third-instar larvae were transferred into Schneider’s media containing 10 µg/mL Pum and incubated in a nutator for 40 min at room temperature. Subsequently, the larvae were snap-frozen in dry ice. The muscle tissues were dissected in ice cold PBS and used to prepare protein lysates for Western blot analyses.

### Lifespan Analysis.

Flies were reared in vials containing standard cornmeal food. Flies were anesthetized using CO_2_ and collected at a density of 20 male flies per vial. All flies were kept at humidified, 12-h on/off light cycle at 25 °C. Flies were flipped into fresh vial every 3 d and the number of dead animals was recorded.

### Cell Lines.

HeLa, U2OS, and HEK293T cells were purchased from ATCC. Cells were cultured under standard tissue culture conditions (1× DMEM [Gibco], 10% FBS, 5% CO_2_, 37 °C).

### Drug Treatments.

HeLa cells were treated with the following drug concentration and times as indicated in the text: Cycloheximide, 50 µg/mL for 4 h; HHT, 5 µM for 10 min; emetine, 100 µM for 15 min; Pum, 100 µM for 15 min; Ans, 0.19 µM for 30 min. For drug treatment in *Drosophila*, adult flies were raised in standard fly food supplemented with 0.25 µg/mL of emetine, or 3 mM Ans for 7 d. To provide chloroquine to flies, 7-d-old flies were first starved for 6 to 8 h. Then a 2 mM chloroquine solution made in 100 mM sucrose was provided to the starved flies via soaked Kimwipe paper for 3 to 4 d. Ten- to 11-d-old flies were dissected to evaluate the level of APP.C99 protein expression in thoracic muscle by Western blot.

### Translation Stalling Reporter Assays.

Analysis of translation readthrough at K20 stall sequences using stalled vs. nonstalled reporter constructs (GFP-P2A-K20-P2A-RFP vs. GFP-P2A-K0-RFP or GFP-P2A-FLAG-K20-P2A-mKate2 vs. GFP-P2A-K0-mKate2) was performed essentially as previously described (12). The pmGFP-P2A-K0-P2A-RFP (#105686) and pmGFP-P2A-K(AAG)20-P2A-RFP (#105689) plasmids were purchased from Addgene. HeLa cells were transfected with K0 and K20 reporters for 24 h, and thereafter NSP1-WT and NSP1-KH were cotransfected for another 36 h. Cells were lysed and processed for immune blot assay. Translation read-through of stall sequence was analyzed by calculation of RFP and GFP ratio. For analysis of GFP-P2A-FLAG-K20-P2A-mKate2 vs. GFP-P2A-K0-mKate2 reporters, HEK293T cells were transfected with reporter constructs using polyethylenimine (1 mg/mL) for 2 d, followed by transfection with Nsp1 plasmid with or without ZNF598 or ASCC3 knockdown. Cellular GFP, FLAG and mKate2 proteins were measured by Western blot analysis.

### Quantification and Statistical Analysis.

Statistical analysis was performed using GraphPad Prism 8 (Windows v8, GraphPad Software). Student’s *t* test and one-way ANOVA test with Tukey’s post hoc test were used for statistical evaluation. All data are represented as mean ± SD, with *P* < 0.05 being considered statistically significant. *P < 0.05, **P < 0.01, ***P < 0.001, ****P < 0.0001.

## Supplementary Material

Supplementary File

## Data Availability

All study data are included in the main text and *SI Appendix*.
