# Supplementary material for: Prevention of ribosome collision-induced neuromuscular degeneration by SARS CoV-2–encoded Nsp1
Source: Proc Natl Acad Sci U S A. 2022 Sep 28;119(42):e2202322119. doi: 10.1073/pnas.2202322119 (PMC9586304; doi:10.1073/pnas.2202322119)
Supplement: Supplementary File [file pnas.2202322119.sapp.pdf]

PNAS

Supplementary Information for

Prevention of ribosome collision-induced neuromuscular degeneration by SARS CoV-2-encoded Nsp1

Xingjun Wang, Suman Rimal, Ishaq Tantray, Ji Geng, Sunil Bhurtel, Tejinder Pal Khaket,

Wen Li, Zhe Han, and Bingwei Lu

Email: bingwei@stanford.edu

This PDF file includes:

Supplemental methods

Figures S1 to S6

Supplementary figure legends

Supplemental Methods

***Drosophila* genetics**

The *UAS-SARS-CoV-2* viral protein transgenic fly lines *UAS-Nsp1, Nsp2, Nsp3, Nsp6, Orf3a, Orf3b, Orf6, Orf7a, Orf7b, Orf8, Orf9b* and *Orf10* were described before (1). The following flies were obtained from the Bloomington *Drosophila* Stock Center: *elav-GAL4* (8765), *UAS-APP.C99* (33783), *UAS-APP* (6700), *UAS-APP;UAS-BACE* (33798), *UAS-LAMP1-GFP* (42714), *UAS-ABCE1-RNAi* (57740), *UAS-ZNF598-RNAi* (61288), *UAS-eRF1-RNAi (67900), UAS-ATG1-RNAi (16133);, UAS-STING-RNAi (31565)*. We obtained *UAS-Clbn-RNAi* (v103351) and *UAS-AKT-RNAi (v103703)* from Vienna *Drosophila* Stock Center. We obtained *UAS-ABCE1* (F001097), *UAS-ZNF598* (F001909), *UAS-Pelo* (F003036) from FLYORF. Drs. T. Littleton provided *MHC-GAL4*, S. Birman provided *TH-GAL4*, Eric Baehrecke provided *UAS-ATG1*, Fen-biao Gao provided *UAS-GR80*, Mel Feany provided *UAS-tau*, Jongkyeong Chung provided *PINK1-B9*, E. Hafen provided *UAS-dFoxo*. *UAS-Q82-YFP* was generated as follows: The Q82-YFP cDNA (a gift from Dr. Richard Morimoto) was cloned into *pUAST* vector, and the resulting *pUAST-87Q-YFP* construct was injected into *w-* embryos to generate transgenic lines. The indicated *UAS* RNAi and OE fly lines were crossed to *Mhc-Gal4* or *elav-Gal4* driver lines for muscle or pan-neuronal expression, respectively. To make the *UAS-Nsp1-KH* transgenic flies, *Nsp1-KH* cDNA with a C-terminal Flag tag amplified from the pcDNA-Nsp1-KH plasmid (Addgene 164522) by PCR was cloned into the *pUAST* vector. Sequence-confirmed construct DNA was injected into embryos by Bestgene to make transgenic flies.

Climbing activity and wing posture assays

Around 10-20 male flies were transferred to a clean plastic vial. The flies were allowed to get accustomed to the new environment for 3-4 min and subsequently measured for bang-induced vertical climbing distance. The performance was scored as percentage of flies crossing the 8 cm mark within 12 seconds. Each experiment was performed ≥ 4 times. To assay wing posture, cohorts of flies raised at 25 or 29 degrees at the indicated ages were visually for straight, held-up, or droopy wing postures. The number of flies with normal (straight) or abnormal (held-up or droopy) wing postures were counted and quantified as the percentage of the total number of flies.

Extraction of fly proteins for western blot analysis

Around 5 fly thoraces or 10 fly heads were homogenized in 80 μl of either regular lysis buffer (50 mM Tris-HCl, 150 mM NaCl, 1% Triton X100, protease inhibitors) or Urea lysis buffer (6 M Urea, 50 mM Tris-HCl, 150 mM NaCl, 0.1% Triton X100, protease inhibitors) on ice. Samples were homogenized using a hand-held mechanical homogenizer for 30 secs. The homogenized samples were incubated on ice for 30 mins before centrifuging at 15000 rpm for 20 mins at 4°C. 30 μl of supernatant was mixed with 10 μl of 4x Lammaelli buffer (BioRad #161-0747) and boiled for 5 mins at 100°C. The protein lysate was cooled, centrifuged and loaded onto 4-12% Bis-Tris gel (Invitrogen #NP0321) or 16% Tricine gel (Invitrogen #EC66955) with 1x MES (Invitrogen #NP0002) as running buffer. For analysis of C-I30-u in *PINK1* mutant flies, a home-made gel system was used as described previously (2).

Aversive taste memory

Drosophila taste memory assay was performed as described previously (3). Briefly, one-week old flies were starved for 12-18h in an empty vial on wet Kimwipe paper before test. Flies were anesthetized on ice and fixed on a glass slide by applying nail polish to their wings. 10-15 flies were used for each set of experiment. Flies were then incubated in a humid chamber for 2 h to allow recovery from the procedure. In the pretest phase, flies were presented with 500 mM sucrose stimuli (attractive tastant) to their legs using Kimwipe wick. Flies that showed positive proboscis extension to the stimulus were used for the next phases. In the second training phase, flies were presented with 500 mM sucrose stimuli at their legs while being simultaneously punished by 10 mM quinine (aversive tastant) applied to their extended proboscis. Training was repeated 15 times for each fly. The last phase is the test phase where the flies were given 500 mM sucrose at their legs at different time intervals (0, 5, 15, 30, 45, and 60 min), and proboscis extension was recorded. Each experiment was carried out ≥ 4 times.

Analysis of gene expression by RT-qPCR

We used TRIzol (Invitrogen) to extract mRNA from fly thorax and iscript cDNA synthesis kit (Bio–Rad) to synthesize cDNA. Real time quantitative PCR (RT-qPCR) was performed using SYBR Green. The sequences of RT-qPCR primers we used are as follows:

AKT forward: GCTATGACGCCATCTGAAC

AKT Reverse: CGCCGCTGCTATTACAAG

ATG1 forward: GCCAGCTCCATCGAAAATAACC

ATG1 Reverse: GCGGCGCAGCAGGCACAG

ATG8a Forward: CCAATACAAGGAGGAGCAC

ATG8a Reverse: AGGAAGTAGAACTGACCGA

mTOR Forward: ACCGATGACGAGGAGAATG

mTOR Reverse: AGCGAAGATACTGTTCAATGG

APP.C99 Forward: TCATTGGACTCATGGTGGGC

APPC.99 Reverse: TCTGCTGCATCTTGGACAGG

Tubulin Forward: CACACCACCCTGGAGCATTC

Tubulin Reverse: CCAATCAGACGGTTCAGGTTG

eRF1 Forward CGACAAGCCCAACATTGC

eRF1 Reverse: GTTTTCCCCACCATACGA

ABCE1-Forward: CGCTGCTACGGTGTGGTTAC

ABCE1-Reverse: GGATCTCGGAGTCGCTGAAG

Protein extraction from cultured cells and western blotting

HeLa or U2OS cells were transfected with the respective plasmids. Cells were washed with 1X PBS 30h post transfection and lysed in lysis buffer (50 mM Tris-HCl, 150 mM NaCl, 1% Triton X100, protease inhibitors), followed by centrifugation at 13000 rpm for 20 mins at 4°C. Protein concentration was measured using the Bradford method. The supernatant was then mixed with 4x protein loading buffer and loaded onto either 4-12% bis-tris gels using MES as running buffer or on 16% Tricine gel and immunoblotted onto PVDF membranes. The membranes were blocked with blocking buffer (5% BSA in TBST) and incubated with following primary antibodies (Anti-Flag, Sigma-Aldrich F1804, 1:2000;

Anti-GFP, ProteinTech 66002, 1:1000; anti-RFP, anti-mKate2, Invitrogen TagRFP Polyclonal Antibody, R10367; Anti-Actin, Sigma-Aldrich A2228, 1:500; 6E10, Bio Legend 803001, 1:1000; Anti-Myc, ProteinTech 16286, 1:1000; Anti-Nsp1, Cell Signaling #57896, 1:200; Anti-ABCE1, abcam (ab32270), 1:1000; Anti-ZNF598, GeneTex GTX119245, 1:250; Anti-Rack1, Santa Cruz sc-17754, 1:1000; Anti-RSAD2, Proteintech 28089-1-AP; anti-JUN, Proteintech 24909-1-AP; anti-p-JUN, Proteintech 28891-1-AP; anti-JNK, Proteintech 24164-1-AP; anti-p-JNK, Cell Signaling #9251). Goat anti-Rabbit IgG HRP, Santa Cruz sc2004 or Goat anti-Mouse IgG-HRP, Santa Cruz sc2005 antibodies were used for detection at 1:10000 dilution. Special steps were taken during SDS PAGE to better resolve the different APP.C99 species as described before (4). For quantification of western blot data, signal intensity was measured and calculated using NIH Image J.

Immunohistochemistry

Immunostaining of adult fly muscle was performed as previously described (5). Briefly, fly thoraxes were dissected and fixed with 4% paraformaldehyde (Electron Microscopy Sciences, cat. no. 15710) in phosphate buffered saline and 0.3% Triton X-100 (PBS-T). Tissues were washed three times with PBS-T and then incubated for 30 min at room temperature in blocking buffer: 0.5% goat serum in PBS-T. The indicated primary antibodies (anti-Ubiquitin, Abcam ab140601, 1:1000; anti-Rab5, Abcam ab31261, 1:1000; anti-P62, Abcam ab178440, 1:1000; 6E10, Bio legend 803001, 1:1000; anti-LAMP1, DSHB 1D4B, 1:100) were added and samples were incubated overnight at 4 °C. The samples were washed three times with PBS-T and subsequently incubated with the indicated secondary antibodies (Alexa Flour 488 (A32723), Alexa flour 594 (A11036),

Invitrogen, 1:200) for 4h at 4 °C. After washing three times with PBS-T, samples were mounted in slow fade gold buffer (Invitrogen).

Immunostaining of adult brains was performed as previously described (4). Briefly, adult fly brains were dissected and fixed on ice for 30-45 min in fixing buffer (940 µl of 1% PBS-T and 60 µl of 37% formaldehyde). Tissues were washed three times in 0.1% PBS-T and blocked overnight at 4°C in blocking buffer (1 ml 1x PBS, 0.1% Triton-X, 5 mg/ml BSA). Following incubation at 4°C for 16 h with the indicated primary antibodies (anti-TH, Pel-Freez P40101-150, 1:1000; anti-Ubiquitin, Abcam ab140601, 1:1000; 6E10, Bio legend 803001, 1:1000; anti-Rab5, Abcam ab31261, 1:1000; anti-P62, Abcam ab178440, 1:1000; Nsp1, Cell Signaling #57896, 1:200; ATP5A1 monoclonal antibody (15H4C4) from Thermo Fisher Catalog # 43-9800, 1:1500), tissues were washed three times with 0.1% PBS-T and subsequently incubated with the appropriate secondary antibodies [Alexa Flour 488 (A32723), Alexa flour 594 (A11036), Invitrogen, 1:200], or F-actin attaining with Alexa Fluor™ 647 Phalloidin from Thermo Fisher (Catalog number: A22287; 1:1000) for 4h at 4°C. Samples were washed three times with 0.1% PBS-T and finally mounted in slow fade gold buffer (Invitrogen) and viewed using a Leica SP8 confocal microscope.

Immunostaining of cultured cells

For immunostaining of cultured cells, HeLa or U2OS cells on coverslips were washed with PBS twice and fixed in 4% paraformaldehyde/PBS solution for 15 mins at RT. Cells were washed repeatedly with 1x PBS prior to incubating with PBS containing 0.1% Triton X-100 for 20 mins. Cells were then incubated in blocking buffer (1x PBS, 0.1% Triton X100,

2% BSA) for 30 mins. After blocking, desired primary antibodies (Anti-cGAS, Proteintech 26416-1-AP; 6E10, BioLegend 803001; Anti-Puromycin, Millipore MABE343; mOC78, Gift from Dr. Glabe) were added to the blocking buffer at 1:1000 concentration and cells were incubated with the antibody solution overnight. The following day, after washing with 1x PBS the following day, cells were incubated in appropriate secondary antibodies for 1 hr. Cells were washed again and the coverslips were mounted on slides using DAPI-containing mounting medium.

For analysis of cGAS distribution in Nsp1 transfected cell with RQC manipulation, ZNF598 KO U2OS cells and ASCC3 KD U2OS cells were transfected with NSP1 and RFP at the ratio of 3 to 1 for two days before harvesting. All cells were harvested and fixed with 3% paraformaldehyde in PBS for 10 minutes at room temperature. This was followed by permeabilization with 0.5% Triton X-100 for 15 minutes. Cells were washed with 1 ml PBS twice and blocked with PBS containing 5% goat serum for 30 minutes at room temperature. Primary antibodies (cGAS) were diluted in blocking buffer (PBS containing 5% goat serum) and incubated in cold room overnight. Cells were washed with 1 ml PBS and incubated with secondary antibody in blocking buffer for 1 h at room temperature. After the final wash cells were mounted on glass slides with antifade mounting medium (Vector Laboratories, H-1700). Images were captured with Leica Confocal Microscope.

Plasmid transfections and siRNA knockdown

Cell transfections were performed by using Lipofectamine 3000 (cat#: L3000015, Invitrogen), and siRNA knockdown experiments were performed using Lipofectamine RNAiMAX reagent (cat#: 13778150, Invitrogen), according to manufacturer's

instructions. For plasmid transfection, cells were plated at 70% confluency the day before in DMEM without antibiotics. On the day of transfection, plasmid DNA and lipofectamine reagent were individually mixed in OptiMEM such that the plasmid/reagent = 1:3. After incubation of 10 mins at room temperature, the plasmid-reagent mixture was added dropwise to the cells. Medium was changed the following day and cells were analyzed 36-48h post transfection. For siRNA treatments, a similar protocol was followed with the final concentration of siRNA at 450 pmol for a 10cm dish (for a 10cm dish: 10 µg of DNA and 20 µl of Lipofectamine 3000, for 6cm dish: 3-5 µg of DNA and 5µl of Lipofectamine 3000 was added). For siRNA and plasmid co-transfection, cells were first treated with siRNA. 24h post transfection with siRNA, medium change and plasmid DNA transfection were carried out. For analysis of the effect of Nsp1 on APP and APP.C99 expression, NSP1 and APP/APP.C99 plasmids mixed at 2:1 ratio were co-transfected in HeLa cells. After 48 hrs, cell lysates were prepared and processed for western blot analysis. For analysis of effect on Nsp1 by RQC factor RNAi, HeLa cells were first transfected with ABCE1 (Stealth RNAi™ siRNA, Invitrogen HSS109285), eRF1 (Stealth RNAi™ siRNA, Invitrogen HSS103392), eIF3E (Stealth RNAi™ siRNA, Invitrogen HSS179956), or RACK1 (Stealth RNAi™ siRNA, Invitrogen HSS115921, Invitrogen) siRNA for 24 hrs. Thereafter, C99 and NSP1 plasmids mixed at 1:2 ratio were co-transfected for 36 hrs. Cell lysates were prepared and western blot analysis was performed. Nsp1-WT and Nsp1-KH mutant plasmids were obtained from Addgene (141255 and 164522).

Puromycin labeling of ribosome stalled newly synthesized proteins

Puromycin labeling of stalled NPCs was performed as described (4). Briefly, Hela cells were seeded on coverslips in a 6-well plate and transfected with pCAX-C99 (Addgene #30146). After 48 hr transfection, cells were treated with HHT for 5 minutes, thereafter Puromycin (50μg) and Emetine (100μg) were added and cells were incubated for 5-7 minutes. After this, cells were permeabilized by 0.02% digitonin in Permeabilization buffer (50 mM Tris-HCl, pH7.5, 5 mM MgCl2, 25 mM KCl, 355 mM cyclohexamide, 10 units RNAseOut and 0.02% digitonin) for 2 minutes. Permeabilized cells were washed twice with washing buffer (permeabilization buffer without digitonin) and fixed in 4% paraformaldehyde for 30 minutes. The Permeabilization and washing steps were performed in ice-old buffers. Cells were immunostained with the amyloid conformation-specific antibody mOC78 that recognizes aggregation-prone APP.C99 (4) and Puromycin antibody and observed under the confocal microscope.

Sucrose gradient analysis of ribosomes

For each experimental condition, one 10 cm plate of cells at around 80% confluency were first washed with ice-cold PBS and harvested by scraping. After sedimentation at 4°C at 5000 rpm for 3 min, cell pellets were resuspended in 200 μl of 1xRNC buffer containing 0.01% digitonin, 1x protease inhibitor cocktail (EDTA-free cOmplete from Roche) and 1mM DTT. After 15 min incubation on ice, cells were disrupted using a pre-chilled 26G needle appended to 1 mL syringe. Lysates were clarified by 15 min centrifugation at 15,000 g at 4°C. Lysate containing 150 μg of total RNA was loaded onto a 10%–50% analytical sucrose gradients (2 ml) and spun for 30 min at 55,000 rpm in TLS-55 rotor at 4°C using slowest acceleration and deceleration settings. Eleven fractions of 200 μl were collected

manually from the top of the gradient. Protein was precipitated from the solution using a final concentration of 20% Trichloroacetic acid (TCA). The resulting pellets were washed with 10% TCA, followed by 100% acetone, and dried. Samples were resuspended in Laemmli sample buffer containing β-mercaptoethanol, boiled at 95°C for 5 min, and subjected to western blot analysis.

Supplemental references

1. J. Y. Zhu, J. G. Lee, J. van de Leemput, H. Lee, Z. Han, Functional analysis of SARS-CoV-2 proteins in Drosophila identifies Orf6-induced pathogenic effects with Selinexor as an effective treatment. *Cell Biosci* **11**, 59 (2021).
2. Z. Wu *et al.*, MISTERMINATE Mechanistically Links Mitochondrial Dysfunction with Proteostasis Failure. *Mol Cell* **75**, 835-848 e838 (2019).
3. P. Masek, K. Worden, Y. Aso, G. M. Rubin, A. C. Keene, A dopamine-modulated neural circuit regulating aversive taste memory in Drosophila. *Curr Biol* **25**, 1535-1541 (2015).
4. S. Rimal *et al.*, Inefficient quality control of ribosome stalling during APP synthesis generates CAT-tailed species that precipitate hallmarks of Alzheimer's disease. *Acta Neuropathol Commun* **9**, 169 (2021).
5. S. Li *et al.*, Quality-control mechanisms targeting translationally stalled and C-terminally extended poly(GR) associated with ALS/FTD. *Proc Natl Acad Sci U S A* **117**, 25104-25115 (2020).

Figure S1

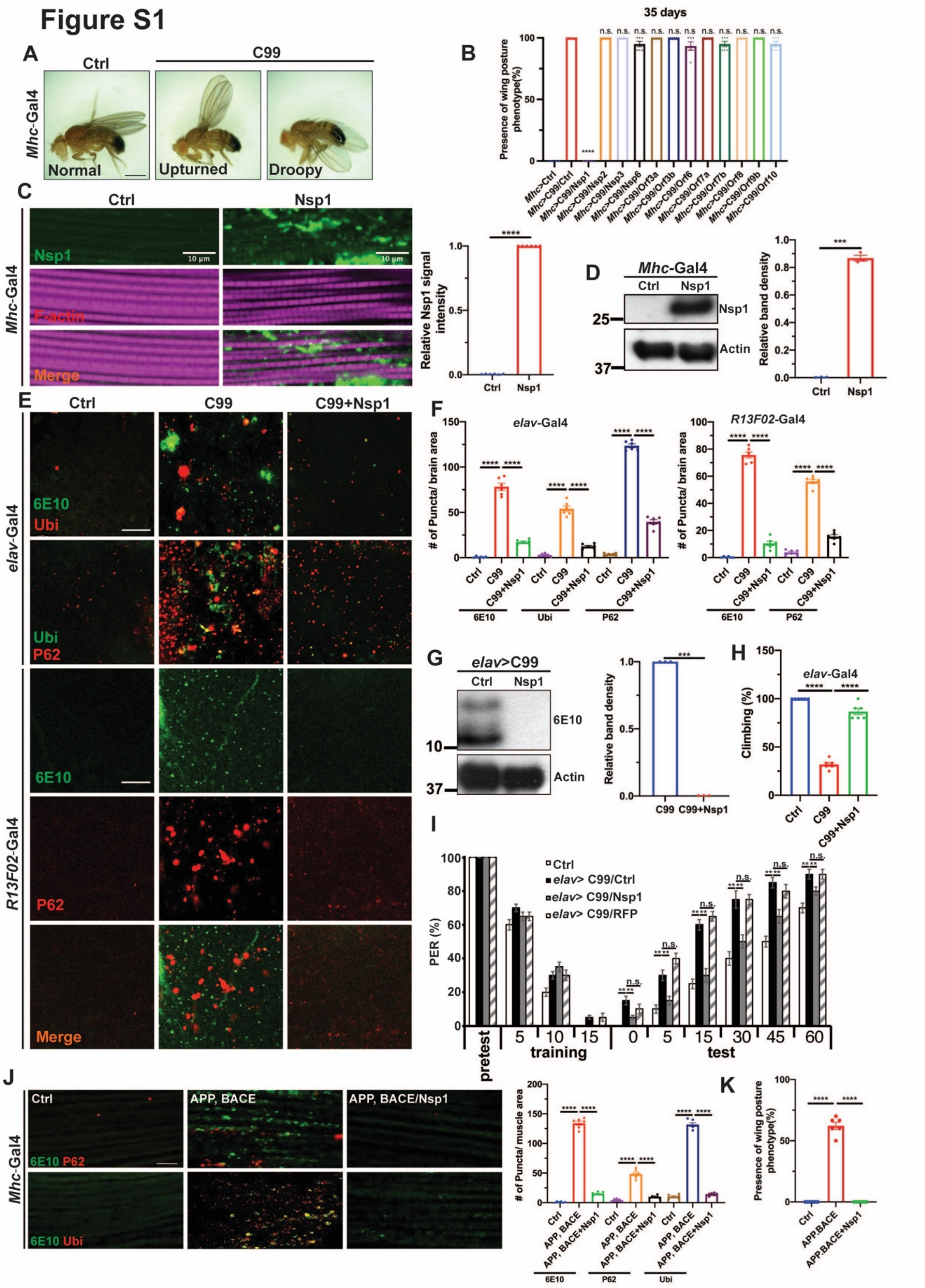
A
Ctrl
C99
Mhc-Gal4
Normal
Upturned
Droopy
B
35 days
Presence of wing posture phenotype(%)
C
Ctrl
Nsp1
Mhc-Gal4
F-actin
Merge
Relative Nsp1 signal intensity
D
Actin
Relative band density
E
C99+Nsp1
elav-Gal4
6E10
Ubi
P62
R13F02-Gal4
F
# of Puncta/ brain area
G
elav>C99
H
Climbing (%)
I
elav> C99/Ctrl
elav> C99/Nsp1
elav> C99/RFP
PER (%)
pretest
training
test
J
APP, BACE
APP, BACE/Nsp1
# of Puncta/ muscle area
K

Fig. S1. Rescue of APP/APP.C99-induced neuromuscular defects by Nsp1 in *Drosophila.* (**A**) Images showing abnormal wing postures in *Mhc>APP.C99* flies. (**B**) Quantification of wing posture defect in *Mhc>APP.C99* flies co-expressing various SARS-CoV-2 encoded viral proteins aged at 35 days (n=6 per group). (**C**) Immunostainings and quantification (n=6) showing Nsp1 protein expression in the muscle in *Mhc>Nsp1* flies. (**D**) Immunoblots and quantification (n=3) showing expression of Nsp1 in *Mhc>Nsp1* flies. (**E**, **F**) Immunostainings and quantification (n=6) showing effect of pan-neuronal (driven by *elav-Gal4*) or mushroom body neuron-specific (driven by *R13F02-Gal4*) expression of Nsp1 on APP.C99-induced proteostasis failure. (**G**) Immunoblots and quantification (n=3) showing effect of Nsp1 on APP.C99 protein level in *elav>APP.C99* fly brain. (**H**) Quantification (n=6) showing effect of Nsp1 on climbing activity in *elav>APP.C99* flies. (**I**) Effect of Nsp1 co-expression on the aversive taste memory deficit in *elav>APP.C99* flies (n=15). (**J**) Immunostaining and quantification (n=6) showing effect of Nsp1 on proteostasis in *Mhc>FL-APP/BACE* fly muscle. (**K**) Quantification (n=6) of wing posture defect in *Mhc>FL-APP/BACE* flies co-expressing Nsp1. Error bars, ± SEM; $^{*}p< 0.05$, $^{**}p<0.01$, $^{***}p<0.001$, $^{****}p<0.0001$, n.s: non-significant, in Student's *t*-tests and one-way ANOVA tests. Scale bars: 1 mm (**A**), 10 µm (**C**), 8 µm (**E**), 20 µm (**J**).

Figure S2

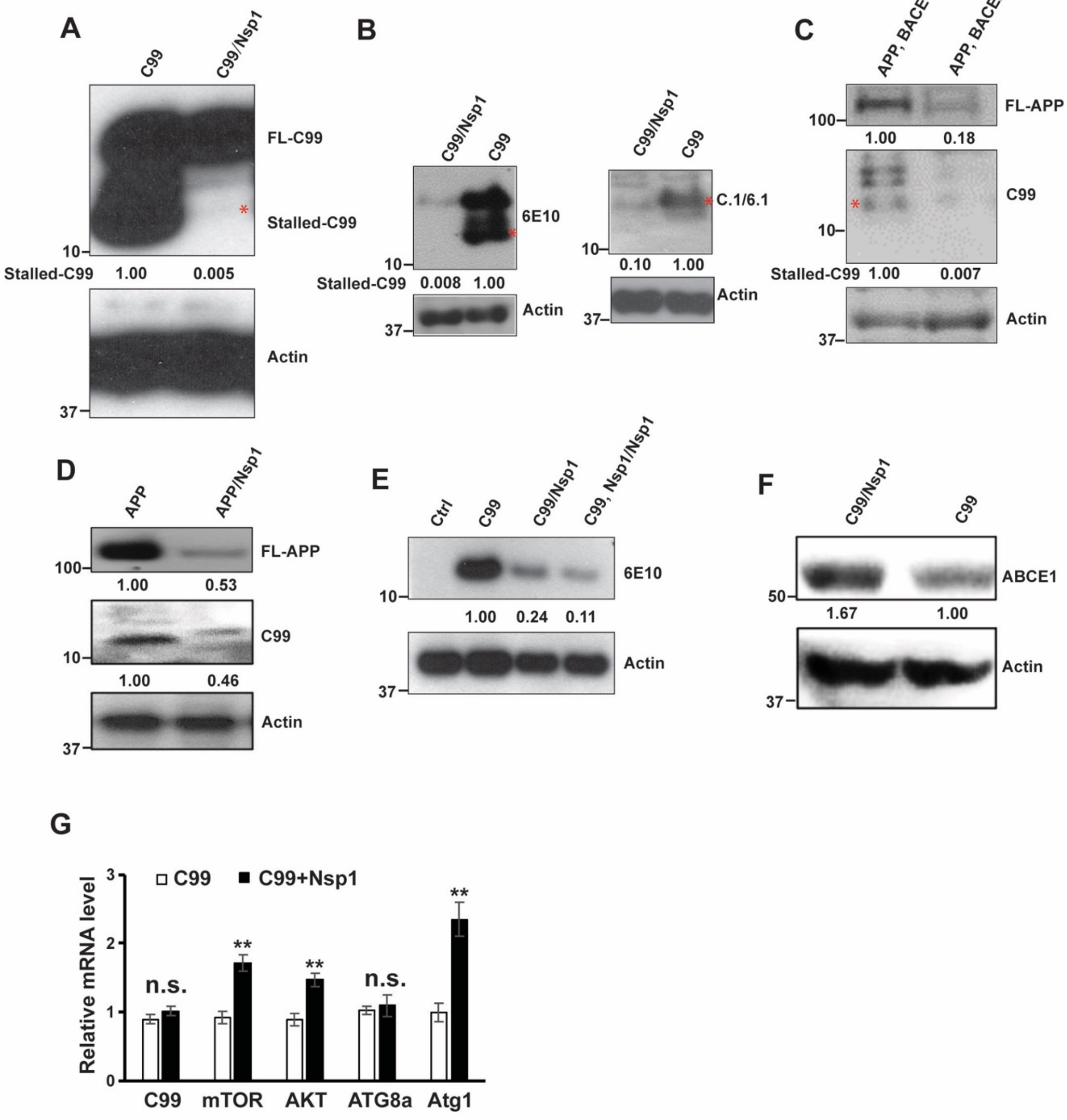
A
C99
C99/Nsp1
FL-C99
Stalled-C99
10
Stalled-C99 1.00 0.005
Actin
37
B
C99/Nsp1
C99
6E10
10
Stalled-C99 0.008 1.00
Actin
37
C99/Nsp1
C99
C.1/6.1
10
0.10 1.00
Actin
37
C
APP, BACE
APP, BACE/Nsp1
FL-APP
100
1.00 0.18
C99
10
Stalled-C99 1.00 0.007
Actin
37
D
APP
APP/Nsp1
FL-APP
100
1.00 0.53
C99
10
1.00 0.46
Actin
37
E
Ctrl
C99
C99/Nsp1
C99, Nsp1/Nsp1
6E10
10
1.00 0.24 0.11
Actin
37
F
C99/Nsp1
C99
ABCE1
50
1.67 1.00
Actin
37
G
C99
C99+Nsp1
Relative mRNA level
3
2
1
0
n.s.
**
**
n.s.
**
C99
mTOR
AKT
ATG8a
Atg1

Fig. S2. Regulation of APP/APP.C99 protein expression by Nsp1. (**A**) Immunoblots showing effect of Nsp1 on FL-APP and APP.C99 levels in *Mhc>FL-APP/BACE* fly muscle. Values below the blots show relative levels of the indicated protein band in this and other figures. (**B**) Immunoblots showing detection of FL-APP.C99 and internally stalled APP.C99 with 6E10 and C.1/6.1 antibodies. (**C, D**) Immunoblots showing effect of Nsp1 on FL-APP and APP.C99 levels in *Mhc>FL-APP/BACE* (**C**) or *Mhc>FL-APP* (**D**) fly muscle. 30 days old flies were used. (**E**) Immunostaining showing effect of one copy or two copies of Nsp1 on APP.C99 level in GMR-Gal4>APP.C99 fly brain extracts. (**F**) Immunoblots showing effect of Nsp1 expression on ABCE1 level in *Mhc>APP.C99* flies. Data are representative of at least two independent repeats in **A**-**F**. (**G**) Quantification of mRNA levels by qRT-PCR in *Mhc>APP.C99* flies with or without Nsp1 co-expression (n=3). **P<0.001, n.s: non-significant, in Student's *t*-test.

Figure S3

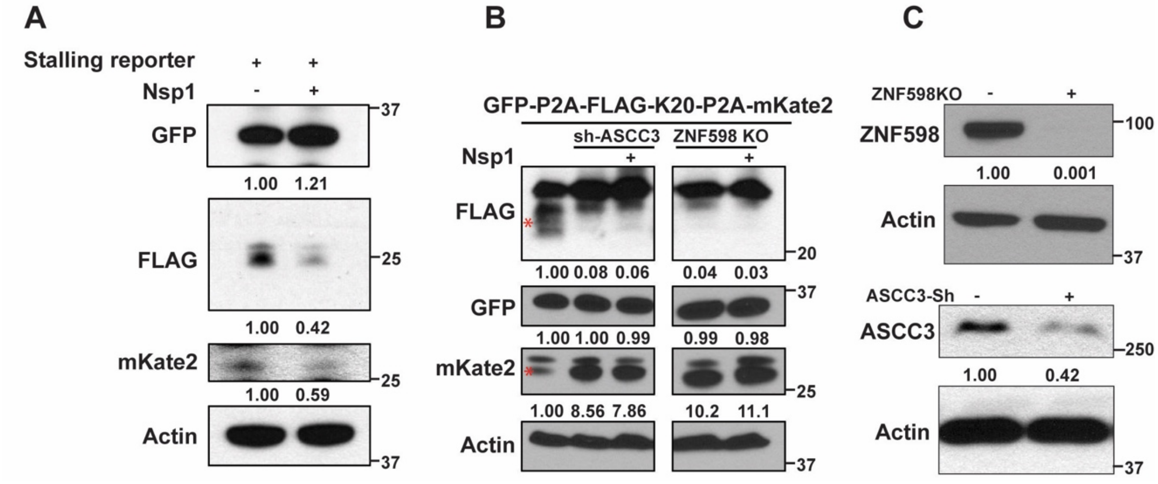
A
Stalling reporter + +
Nsp1 - +
GFP
1.00 1.21
FLAG
1.00 0.42
mKate2
1.00 0.59
Actin
37
25
25
37
B
GFP-P2A-FLAG-K20-P2A-mKate2
sh-ASCC3
ZNF598 KO
Nsp1 + +
FLAG
1.00 0.08 0.06 0.04 0.03
GFP
1.00 1.00 0.99 0.99 0.98
mKate2
1.00 8.56 7.86 10.2 11.1
Actin
20
37
25
37
C
ZNF598KO - +
ZNF598
1.00 0.001
Actin
ASCC3-Sh - +
ASCC3
1.00 0.42
Actin
100
37
250
37

Fig. S3. Nsp1 aborts stalled translation. (**A**) Immunoblots showing effect of Nsp1 on GFP, Flag-K20, and mKate2 expression from the GFP-P2A-Flag-K20-P2A-mKate2 reporter. Actin serves as loading control. (**B**) Immunoblots showing effect of ASCC3 and ZNF598 silencing on GFP, Flag-K20, and mKate2 expression from the GFP-P2A-Flag-K20-P2A-mKate2 reporter in HEK293 cells with or without Nsp1 co-transfection. (**C**) Immunoblots showing knockdown efficiency in ZNF598 KO cells and ASCC3 shRNA transfected cells. Data are representative of at least two independent repeats.

Figure S4

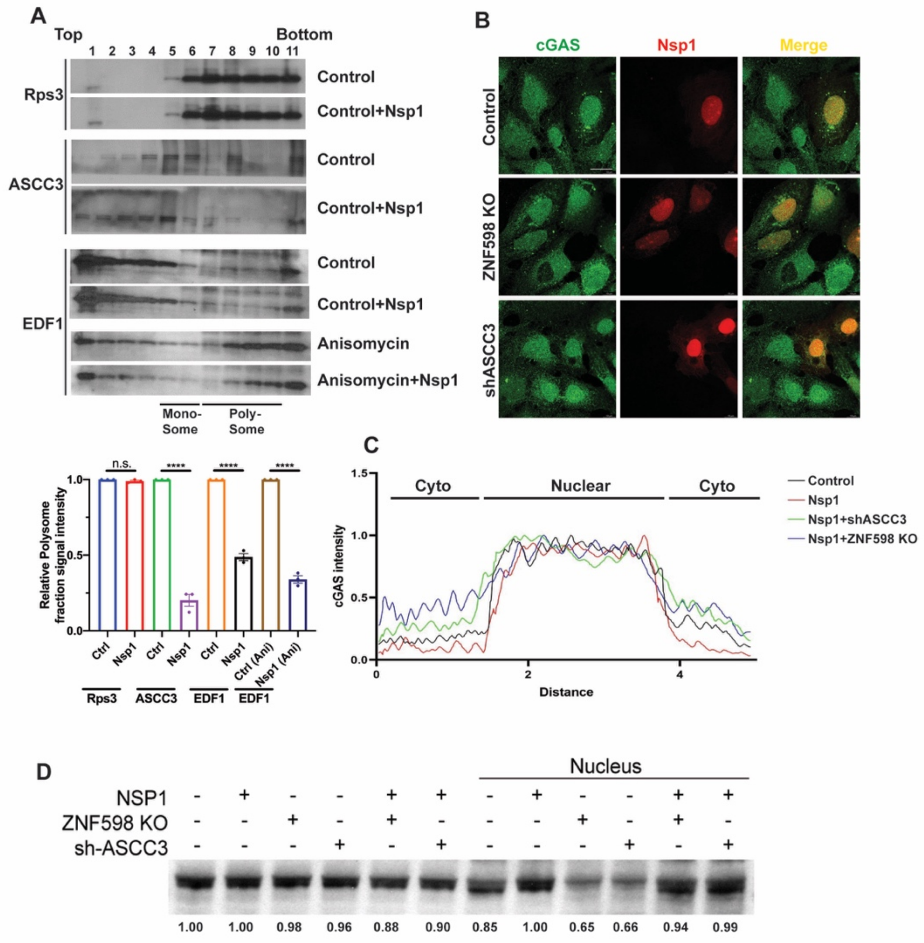
A
Top
Bottom
1 2 3 4 5 6 7 8 9 10 11
Rps3
Control
Control+Nsp1
ASCC3
Control
Control+Nsp1
EDF1
Control
Control+Nsp1
Anisomycin
Anisomycin+Nsp1
Mono-Some
Poly-Some
B
cGAS
Nsp1
Merge
Control
ZNF598 KO
shASCC3
Relative Polysome fraction signal intensity
n.s.
****
Ctrl
Nsp1
Ctrl (Ani)
Nsp1 (Ani)
Rps3
ASCC3
EDF1
C
Cyto
Nuclear
cGAS intensity
Distance
Control
Nsp1
Nsp1+shASCC3
Nsp1+ZNF598 KO
D
Nucleus
NSP1
ZNF598 KO
sh-ASCC3
1.00 1.00 0.98 0.96 0.88 0.90 0.85 1.00 0.65 0.66 0.94 0.99

Fig. S4. Nsp1 resolves collided ribosomes and downregulates cGAS-STING signaling. (**A**) Sucrose gradient analysis of ribosomes showing the effect of Nsp1 on collided ribosomes in HeLa cells. Lysates of control and Nsp1 transfected HeLa cells with or without anisomycin treatment were separated by analytical 10-50% sucrose gradient and proteins in the fractions from top to bottom (1-11) were analyzed by immunoblots for Rps3, ASCC3, and EDF1. Fraction 11 might be contaminated by cell debris and was not included as polysome fraction for analysis. Bar graph shows quantification of relative polysome fraction signal intensity of the indicated proteins in cells with or without Nsp1 expression (n=3). (**B**) Immunostainings showing the effect of Nsp1 on cGAS cytoplasmic vs. nuclear localization in anisomycin-treated U2OS cells with or without silencing of ASCC3 or ZNF598. The red signal in Nsp1 panel represents signal from an RFP reporter co-transfected with Nsp1 at RFP: Nsp1 = 1:2 ratio such that RFP positive cells can be used to help identify Nsp1 transfected cells. (**C**) Representative line scanning of cGAS immunosignal intensity in the cytoplasmic vs. nuclear compartments in cells of the indicated genotypes. (**D**) Immunoblots showing effect of Nsp1 on relative nuclear fraction cGAS level in control and ZNF598 KO or ASCC3 shRNA transfected cells. Data are representative of at least two independent repeats. $^{****}p<0.0001$, n.s: non-significant, in Student's *t*-tests. Scale bar: 18 µm (**B**).

Figure S5

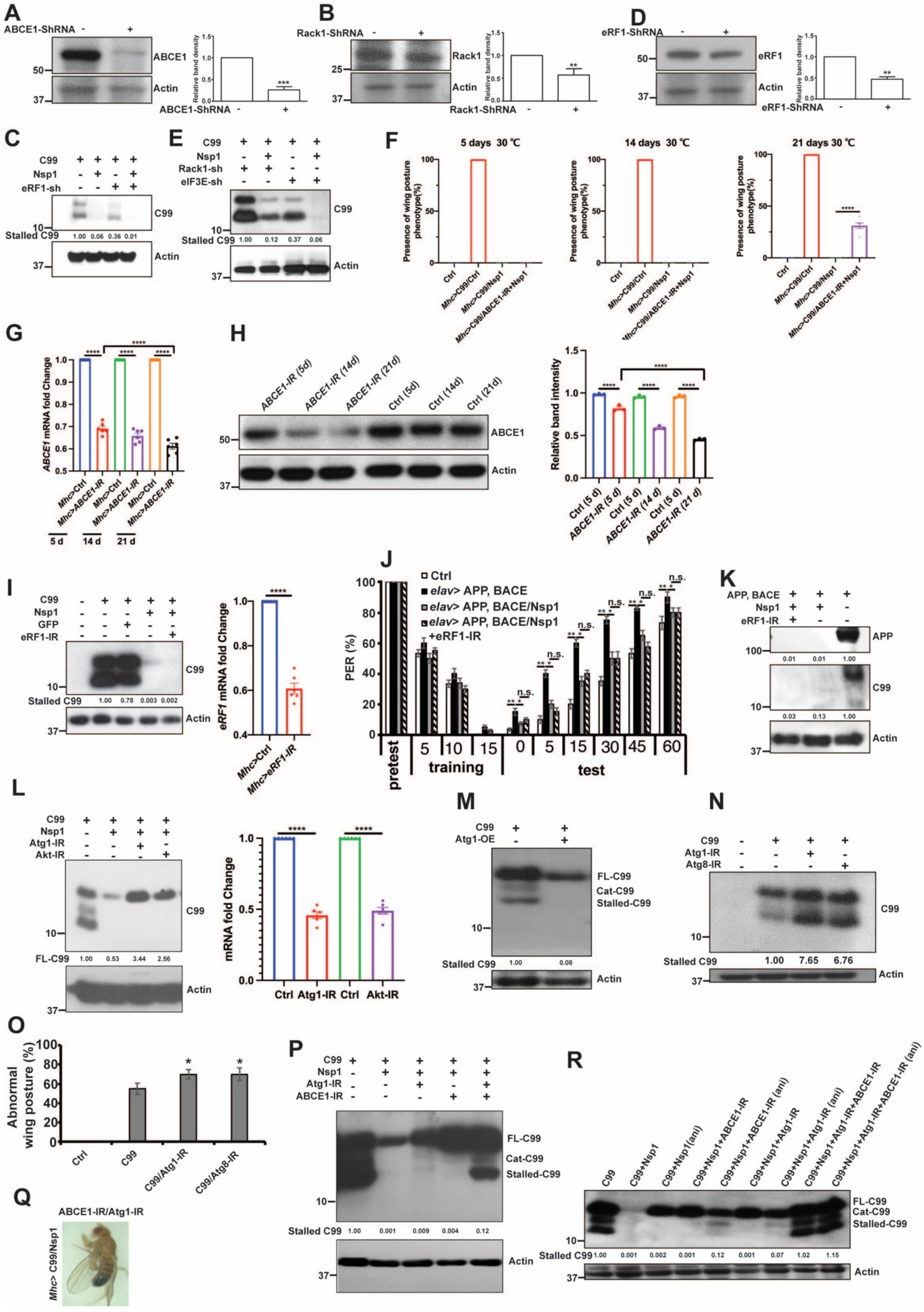

A
ABCE1-ShRNA - +
ABCE1
Actin
50
37
Relative band density
ABCE1-ShRNA - +
B
Rack1-ShRNA - +
Rack1
Actin
25
37
Rack1-ShRNA - +
D
eRF1-ShRNA - +
eRF1
Actin
50
37
eRF1-ShRNA - +
C
C99
Nsp1
eRF1-sh
Stalled C99 1.00 0.06 0.36 0.01
Actin
E
C99
Nsp1
Rack1-sh
eIF3E-sh
Stalled C99 1.00 0.12 0.37 0.06
Actin
F
5 days 30 ℃
14 days 30 ℃
21 days 30 ℃
Presence of wing posture phenotype(%)
Ctrl
Mhc>C99/Ctrl
Mhc>C99/Nsp1
Mhc>C99/ABCE1-IR+Nsp1
G
ABCE1 mRNA fold Change
Mhc>Ctrl
Mhc>ABCE1-IR
5 d
14 d
21 d
H
ABCE1-IR (5d)
ABCE1-IR (14d)
ABCE1-IR (21d)
Ctrl (5d)
Ctrl (14d)
Ctrl (21d)
ABCE1
Actin
Relative band intensity
Ctrl (5 d)
ABCE1-IR (5 d)
ABCE1-IR (14 d)
ABCE1-IR (21 d)
I
C99
Nsp1
GFP
eRF1-IR
Stalled C99 1.00 0.78 0.003 0.002
Actin
eRF1 mRNA fold Change
Mhc>Ctrl
Mhc>eRF1-IR
J
Ctrl
elav> APP, BACE
elav> APP, BACE/Nsp1
elav> APP, BACE/Nsp1 +eRF1-IR
PER (%)
pretest
training
test
K
APP, BACE
Nsp1
eRF1-IR
APP
0.01 0.01 1.00
C99
0.03 0.13 1.00
Actin
L
C99
Nsp1
Atg1-IR
Akt-IR
FL-C99 1.00 0.53 3.44 2.56
Actin
mRNA fold Change
Ctrl
Atg1-IR
Akt-IR
M
C99
Atg1-OE
FL-C99
Cat-C99
Stalled-C99
Stalled C99 1.00 0.08
Actin
N
C99
Atg1-IR
Atg8-IR
Stalled C99 1.00 7.65 6.76
Actin
O
Abnormal wing posture (%)
Ctrl
C99
C99/Atg1-IR
C99/Atg8-IR
P
C99
Nsp1
Atg1-IR
ABCE1-IR
FL-C99
Cat-C99
Stalled-C99
Stalled C99 1.00 0.001 0.009 0.004 0.12
Actin
Q
ABCE1-IR/Atg1-IR
Mhc> C99/Nsp1
R
C99
C99+Nsp1
C99+Nsp1(ani)
C99+Nsp1+ABCE1-IR
C99+Nsp1+ABCE1-IR (ani)
C99+Nsp1+Atg1-IR
C99+Nsp1+Atg1-IR (ani)
C99+Nsp1+Atg1-IR+ABCE1-IR
C99+Nsp1+Atg1-IR+ABCE1-IR (ani)
FL-C99
Cat-C99
Stalled-C99
Stalled C99 1.00 0.001 0.002 0.001 0.12 0.001 0.07 1.02 1.15
Actin

Fig. S5. ABCE1 mediates the effect of Nsp1 on APP/APP.C99 translation and toxicity. (**A**, **B**) Immunoblots and quantification (n=3) showing effects of ABCE1-shRNA and Rack1-shRNA knockdown efficiency. (**C**) Immunoblots showing effect of eRF1 RNAi on the removal of stalled APP.C99 species by Nsp1. (**D**) Immunoblot and quantification (n=3) showing effect of eRF1-shRNA knockdown efficiency. (**E**) Immunoblots showing effect of eIF3E-shRNA on the removal of stalled APP.C99 species by Nsp1. Rack1 RNAi serves as a positive control. (**F**) Quantification showing age-dependent effect of ABCE1 RNAi on the wing posture defect rescued by Nsp1 in *Mhc>APP.C99* flies (n=6). (**G**) RT-PCR analysis of age-dependent ABCE1-RNAi efficiency. (**H**) Immunoblots and quantification (n=3) showing age-dependent effect of ABCE1-RNAi efficiency on protein level. (**I**) Immunoblots showing effect of eRF1 RNAi on the removal of stalled APP.C99 species by Nsp1 in *Mhc>APP.C99* fly muscle. Graph on the right shows RT-PCR analysis of eRF1 RNAi efficacy. (J) Quantification showing effect of eRF1 RNAi on the rescue by Nsp1 of the aversive taste memory deficit in *elav>APP/BACE* flies (n=15). (**K**) Immunoblots showing effect of eRF1 RNAi on the level of FL-APP and APP.C99 removed by Nsp1 in *elav>FL-APP/BACE* fly brain. (**L**) Immunoblot showing effect of ATG1 RNAi and AKT RNAi on the level of stalled APP.C99 species removed by Nsp1 in *Mhc>APP.C99* fly muscle. Bar graph on the right show RT-PCR analysis of ATG1 RNAi and ABCE1 RNAi knockdown efficacy (n=3). (**M, N**) Immunoblots showing effect of Atg1-OE (**M**) or Atg1-RNAi, Atg8-RNAi (**N**) on FL-APP.C99 and stalled APP.C99 levels in Mhc>APP.C99 flies. (**O**) Quantification of effect of Atg1-RNAi or Atg8-RNAi on wing posture defect caused by APP.C99 (n=6). (**P**) Immunoblots showing effect of combined ATG1 RNAi and ABCE1 RNAi on the level of stalled APP.C99 species removed by Nsp1 in *Mhc>APP.C99*

fly muscle. FL-APP.C99, CAT-tailed APP.C99, and internally stalled APP.C99 are indicated. (**Q**) Image showing abnormal wing posture phenotype recovered by combined ATG1 RNAi and ABCE1 RNAi in *Mhc>APP.C99* flies co-expressing Nsp1. (**R**) Immunoblots showing effect of anisomycin treatment on the level of stalled APP.C99 species removed by Nsp1 in the various genetic backgrounds. Error bars, ± SEM; *p< 0.05, **p<0.01, ***p<0.001, ****p<0.0001, n.s: non-significant, in Student's *t*-tests and one-way ANOVA tests. Scale bar: 1 mm (**Q**).

Figure S6

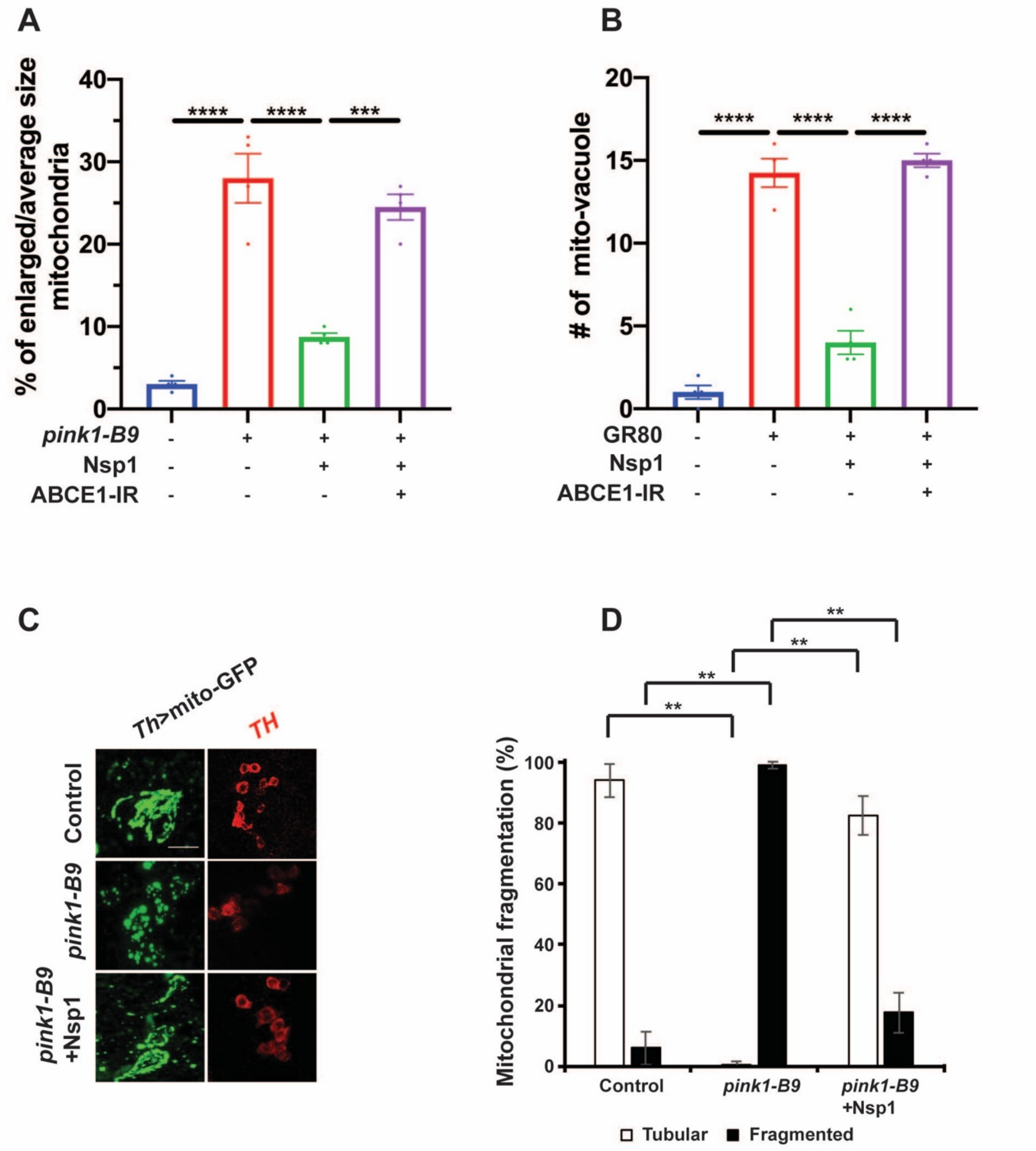

A
% of enlarged/average size mitochondria
40
30
20
10
0
****
****
***
pink1-B9 - + + +
Nsp1 - - + +
ABCE1-IR - - - +
B
# of mito-vacuole
20
15
10
5
0
****
****
****
GR80 - + + +
Nsp1 - - + +
ABCE1-IR - - - +
C
Th>mito-GFP
TH
Control
pink1-B9
pink1-B9
+Nsp1
D
**
**
**
**
Mitochondrial fragmentation (%)
100
80
60
40
20
0
Control
pink1-B9
pink1-B9
+Nsp1
Tubular
Fragmented

Fig. S6. Nsp1 specifically and robustly rescues the neuromuscular degeneration phenotypes in *Drosophila* PD and ALS models. (**A, B**) Quantification of mitochondrial morphology of the images shown in Fig. 6D, E (n=6). (**C**) Immunostainings showing the effect of Nsp1 on DA neuron mitochondrial morphology in $PINK1^{B9}$ mutant fly brains. Mitochondrial morphology was monitored with a mito-GFP reporter driven by *TH-Gal4*. DA neurons in the PPL1 cluster were shown. (**D**) Quantification of data shown in (**C**). The relative abundance of tubular vs. swollen mitochondria was quantified (n=6). **P<0.01, ***p<0.001, ****p<0.0001, in Student's *t*-test. Scale bar: 8 μm (**C**).

Figure S7

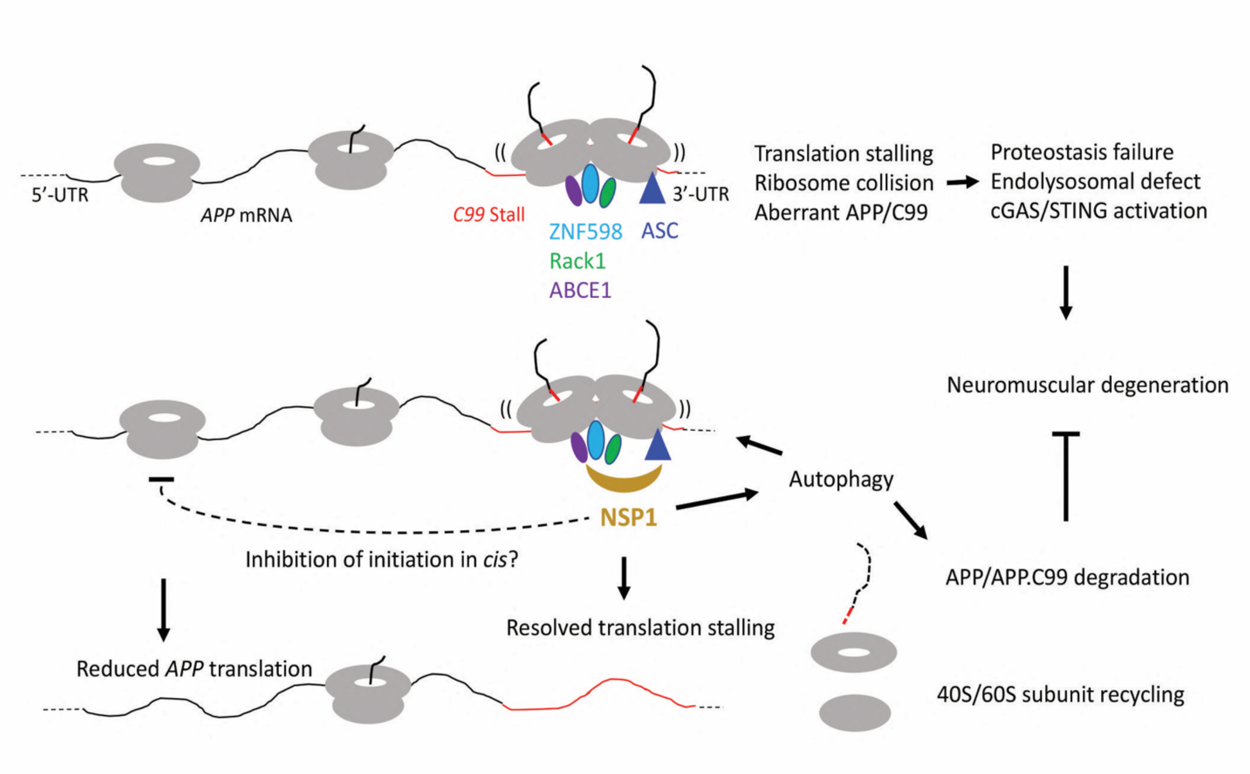

Figure S7. Schematic diagram of the potential mechanisms of Nsp1 action in resolving collided ribosomes and rescuing neuromuscular degeneration

Diagram uses *APP* translation as example to depict the multipronged strategy of Nsp1 in regulating RQC factor recruitment to resolve stalled mRNA translation and restore cellular homeostasis, and the link between inadequate quality control of stalled translation and collided ribosomes and various hallmarks of neurodegenerative diseases, including proteostasis failure, endolysosomal defects, and neuroinflammation.
